# *Vibrio cholerae* cytolysin induces pro-inflammatory and death signals through novel TLR assembly

**DOI:** 10.1371/journal.ppat.1013033

**Published:** 2025-04-04

**Authors:** Shraddha Gandhi, Sindhoora Puravankara, Anish Kumar Mondal, Aakanksha Chauhan, Shashi Prakash Yadav, Kausik Chattopadhyay, Arunika Mukhopadhaya

**Affiliations:** Department of Biological Sciences, Indian Institute of Science Education and Research Mohali, Mohali, Punjab, India; St Jude Children's Research Hospital, UNITED STATES OF AMERICA

## Abstract

*Vibrio cholerae* cytolysin (VCC) is a potent exotoxin secreted by *Vibrio cholerae*, the etiological agent of the severe diarrheal disease cholera. VCC is a membrane-damaging pore-forming toxin by nature, and is well known for its ability to cause host cell death. Using wild type *V. cholerae* and VCC-deleted mutant variant of the bacteria, we show that VCC plays an important role in the inflammatory responses during infection in mice. This observation supports that VCC can function as a pathogen-associated molecular pattern (PAMP). Toll-like receptors (TLRs) are the key initiators of inflammation. Upon ligand recognition, TLR1 and TLR6 generally form heterodimers with TLR2 for triggering pro-inflammatory signals. In the present study, we show that VCC engages novel TLR1/4 heterodimer assembly, and elicits pro-inflammatory responses in both dendritic cells (DCs) and macrophages. Along with TLR1/4, VCC-induced pro-inflammatory response in macrophages also involves TLR2. It has been shown earlier that VCC is implicated in the *V. cholerae*-mediated killing of the immune cells following biofilm formation. Here we show that TLRs play an important role in VCC-mediated killing of DCs and macrophages following *V. cholerae* infection. Interestingly, we find that TLR1/4 signalling is specifically crucial for the VCC-induced inflammatory and death responses in DCs, as well as in mice. Additionally, we observe that similar to DCs and macrophages, TLR1/4-MyD88 play an important role in VCC-mediated inflammatory responses in another crucial immune cell type, neutrophils. Taken together, our study shows novel TLR heterodimer formation, differential recognition of the same ligand by different TLR combination in cell type-dependent manner, and their implications in the context of *V. cholerae* and VCC-induced immune cell death and mortality.

## Author Summary

VCC is an important toxin secreted by *Vibrio cholerae*, the causal agent of human diarrheal disease cholera. VCC forms pores in the host cell membranes, and thus plays crucial role in pathogenesis. In addition to cell lysis via punching holes in the cell membranes, VCC can induce programmed cell death. *V. cholerae* forms biofilm surrounding immune cells and kills them using VCC. Though host-cell killing seems to be the major function of VCC, it can also modulate other responses of the host. Here we show that VCC can induce activation of the major immune cells, such as dendritic cells (DCs), macrophages, and neutrophils in terms of pro-inflammatory responses by engaging novel Toll-like Receptor (TLR) combination. TLR2 generally forms heterodimer with TLR1 or TLR6. However, we observe that VCC employs TLR1/4 heterodimer for the induction of pro-inflammatory responses. Interestingly, for the VCC-mediated pro-inflammatory signals, macrophages, but not DCs, also employ additional receptors, such as TLR2/6. Further, we observe that the novel receptor combination of TLR1/4 heterodimer is critical for the VCC-induced cell death and mortality of mice, suggesting its important role in the pore-independent cell death by VCC.

## Introduction

Bacterial pore-forming toxins (PFTs) form pores in the target cells’ plasma membranes that generally lead to cell death by colloid-osmotic lysis [1]. Additionally, PFTs can kill target mammalian cells by inducing classical/non-classical programmed cell death [2–6]. However, apart from their roles in cell death, PFTs have also been shown to activate a plethora of intra-cellular signalling cascades [2,7]. One such attribute of the PFTs is the modulation of the host’s immune responses [8]. PFTs can effectively serve as pathogen-associated molecular patterns (PAMPs), and get recognized by the pattern-recognition receptors (PRRs), present on the immune cells [9–11]. Many PFTs are reported to be recognized by different Toll-like Receptors (TLRs) present on the immune cells, thereby generating a multitude of signalling cascades resulting in the pro- and/or anti-inflammatory cytokine responses [10,12,13]. Though there are few reports, yet, the role of the PFTs in modulating the host’s immune responses is under-explored, compared to studies exploring their roles in the cell death induction [2].

*Vibrio cholerae* cytolysin (VCC) is a β-barrel PFT (β-PFT) secreted by the Gram-negative bacterial pathogen *V. cholerae* [14,15]. It is secreted by the bacterium in the form of a precursor, termed as pro-VCC, and subsequent proteolytic removal of the pro-domain by the bacterial proteases generates the mature form of the toxin [16]. Monomeric form of mature VCC binds to the target-cell membranes, and then assembles into heptameric, β-barrel pores of approximately 1-2 nm diameter [17,18]. Pore formation in the cell membranes results in the disruption of the membrane permeability barrier function. Additionally, VCC can lead to the induction of caspase-dependent apoptosis in the target cells. VCC can also elicit cell survival response in the target cells through the induction of autophagy [3,19,20]. Further, by acting as a PAMP, VCC can induce pro-inflammatory responses in the innate immune cells, such as monocytes, macrophages, and mast cells [10,21,22].

Being an enteric pathogen, *V. cholerae* colonizes the small intestine and causes the severe secretory diarrheal disease, cholera. Cholera is associated with the microscopic changes in the intestine characterised by the widening of the inter-cellular spaces, and disruption of the apical junctions, resulting in an influx of the immune cells, including neutrophils, mast cells, macrophages, and dendritic cells (DCs) [23–25]. Thus, the toxins secreted by the bacteria in the milieu, such as VCC, would come in contact with the immune cells, resulting in their activation and generation of the pro-inflammatory responses. M cells present in the Peyer’s patches can transcytose luminal antigens, which are then taken up by the nearby DCs. Furthermore, DCs migrating to the lymph nodes sample antigens from the intestinal lumen by forming tight junctions with the intestinal epithelial cells. Therefore, VCC could potentially interact with the plethora of host immune cells, and modulate their functions to shape up the outcome of the host-pathogen interaction process. PFTs, like VCC, can also generate damage-associated molecular patterns (DAMPs) by lysing the host cell membranes. Moreover, the fragmented host cell membranes encompassing the PFT oligomers can act as the DAMP/PAMP. For example, *Streptococcus pneumoniae* pneumolysin (PLY)-containing micro-vesicles/liposomes have been shown to act as the DAMP-like signals to polarize the macrophages [26]. However, there has been very little in-depth exploration regarding the cellular responses generated by the PFT-induced DAMPs, originating from the lysed or dead cells. Consistent with its pore-forming toxin nature, VCC attacks the target cells, and irreversibly converts into the oligomeric assembly in the lipid bilayer of the cell membranes. The oligomeric form of VCC embedded in the fragmented membranes of the lysed cells can also be recognized by the host immune system as a potential DAMP. Therefore, it would be important to study the effects of the pre-formed oligomeric form of VCC as well, along with the monomeric form of the toxin, on the cells of the immune system for generating the inflammatory responses.

Previous studies have suggested that VCC monomer can mediate downstream signals in different innate immune cell types via activation of TLR2, MAPKs and NF-κB pathways, and also through inflammasome activation [22,27,28]. Further, the oligomeric form of VCC, generated in the presence of the lipid bilayer of liposome membranes, has been shown to activate macrophages and monocytes in terms of production of pro-inflammatory mediators (such as nitric oxide (NO), interleukin-6 (IL-6) and tumor necrosis factor-α (TNF-α)) via TLR2/6 heterodimer engagement [10]. TLR2/6 activation by the liposome-embedded VCC oligomers has been shown to trigger activation of the downstream signalling cascades that involve MyD88/IRAK-1/TRAF6 and MAPK, culminating in the activation of the transcription factors NF-κB and AP-1 [10].

Dendritic cells (DCs) are one of the most crucial cell types of the immune system. DCs not only contribute to the pro-inflammatory response generation, but also decide the fate of the adaptive immunity [29,30]. Therefore, interaction of a PAMP or pathogen-derived virulence factor with the DCs can not only regulate the outcome of the innate immune responses, but it may shape up (either in a positive or negative manner) the adaptive immune responses as well. Previous studies have explored the interactions of the mature monomeric form of VCC with macrophages, monocytes, and mast cells. Pre-formed VCC oligomeric form incorporated into the lipid bilayer of liposomes has also been studied for its ability to act as a PAMP to activate macrophages and monocytes [10,21,22]. However, it has not been explored yet whether VCC can induce the activation of DCs.

Considering the fact that a cognate ligand-receptor interaction primarily modulates the intra-cellular signalling, and drives the functional immune responses, in this study, we have investigated the PRRs involved in the VCC recognition in the bone marrow-derived dendritic cells (BMDCs). We have shown that both the monomeric and oligomeric forms of VCC activate the BMDCs leading to the production of pro-inflammatory cytokines. Though both VCC monomer and pre-formed VCC oligomer have so far been shown to induce activation of the innate immune cells via TLR2, in the present study, we have demonstrated that in DCs, both forms of VCC trigger the assembly of a novel TLR1/TLR4 heterodimer to mediate the signalling process. TLR1/4 signalling is propagated via MyD88 adaptor molecule leading to the production of pro-inflammatory cytokines. VCC-mediated activation of DCs also involves the scavenger receptor CD36 (cluster of differentiation 36). We have also found that, though in DCs both the forms of VCC are recognized by TLR1/4 heterodimer, in macrophages they engage both TLR1/4 and TLR2/6. Further, our results indicate that the differential expression of these receptors on DCs and macrophages, at the basal level, as well as upon VCC stimulation, could be the reason for differential recognition of the same ligand by different TLR combinations in the two cell types. We have also observed that VCC could induce activation of neutrophils through TLR1/4/MyD88-mediated signalling. Our study also reveals that TLRs play crucial role in the VCC-induced inflammatory response and mortality in mice, and different TLRs are engaged in the induction of the VCC-mediated death signal in DCs and macrophages.

## Results

### VCC monomer and oligomer can activate BMDCs in terms of the production of pro-inflammatory cytokines

To explore any possible cross-talk between VCC and DCs, in the present study, we wanted to examine whether VCC monomer and its pre-formed oligomeric state assembled in the liposomes (designated henceforth as the VCC oligomer) can induce DC activation. In order to examine the ability of VCC to activate DCs, we assessed the production of pro-inflammatory cytokines, such as IL-6, TNF-α and IL-1β in BMDCs in response to VCC monomer and oligomer. For this, BMDCs were treated with VCC monomer (10 ng/ml) or pre-formed VCC oligomer (1 μg/ml). Additionally, the cells were treated with either buffer or an equivalent amount of only liposomes that was present in the VCC oligomer preparation. Following 24 hours of incubation, cell culture supernatants were analysed for the production of the cytokines using ELISA. We observed that the cytokine levels produced by the cells upon treatment with either buffer or the liposomes were almost negligible compared to those from the VCC monomer or the oligomer-treated cells (S1 Fig). Therefore, buffer-treatment served as the negative control for the experiments with VCC monomer, while treatment with an equivalent amount of liposomes served as the negative control for the experiments with VCC oligomer. In the case of VCC monomer and oligomer treatments, we observed that at even 10 ng/ml concentration, VCC monomer induced significant cytotoxicity (around 70-80%), and ~60% loss of viability in the BMDCs, whereas VCC oligomer, at 1 μg/ml concentration, induced relatively less cytotoxicity (around 30%), and ~30% loss of viability in the BMDCs (S1A-S1B Fig). Whatsoever, we found that both VCC monomer and VCC oligomer treatment led to a significant increase in the production of IL-6 (S1C Fig), TNF-α (S1D Fig), and IL-1β (S1E Fig) in BMDCs, suggesting that both the forms of VCC can activate DCs. However, we observed that the cytokine productions in the VCC oligomer-treated cells were much higher than those in the VCC monomer-treated BMDCs. Due to profound cytotoxicity caused by VCC monomer, even at 10 ng/ml concentration (S1A-S1B Fig), it is possible that it might have compromised the extent of cytokine production by the target BMDCs in response to VCC monomer. In contrast, the pre-formed VCC oligomer, once generated, cannot further execute membrane-damaging pore-forming activity [31]. Therefore, the pre-formed VCC oligomer exhibited significantly reduced cytotoxicity compared to the monomeric form. Due to the reduced cytotoxicity in the target cell population, higher number of residual surviving cells could produce higher pro-inflammatory cytokine responses. Hence, in the subsequent experiments of our present study, we employed the concentrations of both VCC monomer and oligomer that could trigger significant pro-inflammatory cytokine responses, while inducing lesser or minimal cytotoxicity. Accordingly, we used 10 ng/ml concentration of VCC monomer and 1 μg/ml concentration of VCC oligomer for our further experiments with BMDCs. It is important to note that the concentrations used here are lesser/comparable than those used in the previous studies exploring the effects of different PFTs on the immune cells [21,32,33], and are presumably within the physiologically relevant concentration range of the toxin [34–36].

### VCC-induced pro-inflammatory responses in BMDCs are TLR1-dependent but not TLR2-dependent

It has been shown in a previous study that VCC oligomer can be recognized by the TLR2/6 heterodimer in the monocytes and macrophages to generate pro-inflammatory signals [10]. Therefore, at least VCC oligomer would be expected to mediate the activation signal(s) via TLR2/6 in the DCs as well. TLR2 generally forms heterodimer with TLR1 or TLR6, and a PAMP can mediate signalling via either TLR1/2 or TLR2/6 heterodimer. However, it has been shown in the previous studies that *Vibrio parahaemolyticus* outer membrane protein OmpU and *Salmonella* Typhimurium outer membrane protein OmpV can be recognised as PAMPs by both TLR1/2 and TLR2/6 heterodimers, simultaneously [37, 38].

**Fig 1:**
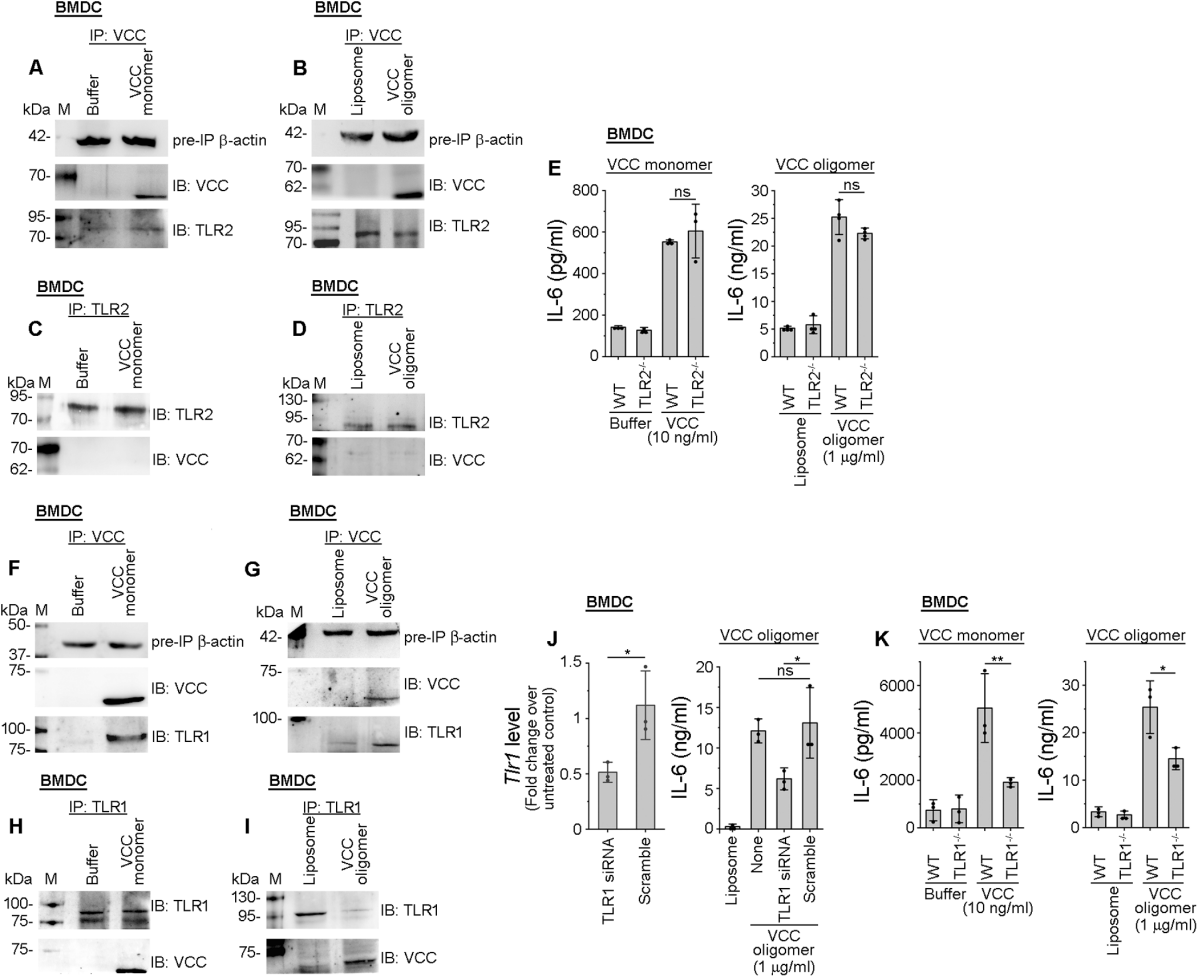
VCC-induced pro-inflammatory responses in DCs are TLR1-dependent but not TLR2-dependent. (A-B) Immunoblot analyses to probe TLR2 co-immunoprecipitation with VCC in BMDCs, treated with VCC monomer (A) or pre-formed VCC oligomer (B). Cells were treated with VCC monomer (10 ng/ml; A) or pre-formed VCC oligomer (1 μg/ml; B) for 30 minutes. Cell lysates were subjected to immunoprecipitation (IP) with anti-VCC antibody, and immunoprecipitated fractions were subsequently probed for TLR2 and VCC by immunoblotting (IB) using anti-TLR2 and anti-VCC antibodies, respectively. β-actin was probed as the pre-IP protein loading control. Buffer and liposome-treated cells served as the negative controls for the experiments with VCC monomer and oligomer, respectively. Data shown here are the representatives of three independent experiments. (C-D) VCC did not co-immunoprecipitate with TLR2 in BMDCs, treated with VCC monomer (C) or pre-formed VCC oligomer (D). BMDCs were treated with VCC monomer (10 ng/ml; C) or pre-formed oligomer (1 μg/ml; D) for 30 minutes. Cell lysates were subjected to immunoprecipitation (IP) with anti-TLR2 antibody, and immunoprecipitated fractions were probed for TLR2 and VCC by immunoblotting (IB) using anti-TLR2 and anti-VCC antibodies, respectively. TLR2 was probed as the protein loading control. Buffer and liposome-treated cells served as negative controls for the experiments with VCC monomer and oligomer, respectively. Immunoblots shown here are the representatives of three independent experiments. (E) No significant decrease in the VCC-induced IL-6 production in BMDCs from TLR2^-/-^ mice, as compared to that in BMDCs from the wild type (WT) mice. BMDCs from wild type (WT) and TLR2^-/-^ mice were stimulated with VCC monomer (10 ng/ml) or pre-formed VCC oligomer (1 μg/ml) for 24 hours. Following the treatments, IL-6 production was monitored by ELISA. Cells treated with buffer and liposomes served as the negative controls for the experiments with VCC monomer and oligomer, respectively. Data shown here are the averages ± SDs from three to four independent experiments. ns, non-significant; p > 0.05; one-way ANOVA with Tukey’s multiple comparison test. (F-G) Immunoblot analyses showing co-immunoprecipitation of TLR1 with VCC in BMDCs, treated with VCC monomer (F) or pre-formed VCC oligomer (G). Cells were treated with VCC monomer (10 ng/ml; F) or pre-formed VCC oligomer (1 μg/ml; G) for 30 minutes. Cell lysates were subjected to immunoprecipitation (IP) with anti-VCC antibody, and immunoprecipitated fractions were subsequently probed for TLR1 and VCC by immunoblotting (IB) using anti-TLR1 and anti-VCC antibodies, respectively. β-actin was probed as the pre-IP protein loading control. Buffer and liposome-treated cells served as the negative controls for the experiments with VCC monomer and oligomer, respectively. Data shown here are the representatives of three independent experiments. (H-I) VCC co-immunoprecipitates with TLR1 in BMDCs, treated with VCC monomer (H) or pre-formed VCC oligomer (I). BMDCs were treated with VCC monomer (10 ng/ml) or pre-formed VCC oligomer (1 μg/ml) for 30 minutes. Cell lysates were subjected to immunoprecipitation (IP) with anti-TLR1 antibody, and immunoprecipitated fractions were probed for TLR1 and VCC by immunoblotting (IB) using anti-TLR1 and anti-VCC antibodies, respectively. TLR1 was probed as the protein loading control. Buffer and liposome-treated cells served as negative controls for the experiments with VCC monomer and oligomer, respectively. Immunoblots shown here are the representatives of three independent experiments. (J) siRNA-mediated knock-down of TLR1 leads to a decrease in IL-6 production in BMDCs, upon treatment of the cells with pre-formed VCC oligomer. Cells were transfected with 75 nM siRNA against TLR1 or non-target control (scramble). Knock-down of TLR1 was confirmed using RT-qPCR after 48 hours of transfection (left panel; showing the fold change in the *Tlr1* level over the untreated untransfected control). After 48 hours, cells were treated with 1 μg/ml pre-formed oligomer, and incubated for 12 hours. Subsequently, the cell supernatant was analysed for IL-6 production by ELISA (right panel). Scramble-transfected cells upon VCC oligomer treatment served as the siRNA control. Liposome-treated cells were also analysed as control. Data shown here are the averages ± SDs from three independent experiments. ns, non-significant; *, p < 0.05; Student’s unpaired t-test (for the TLR1 expression data) and one-way ANOVA with Tukey’s multiple comparison test (for the IL-6 production data). (K) Significant decrease in the IL-6 production in the VCC-treated BMDCs from TLR1^-/-^ mice, as compared to those from the wild type (WT) mice. BMDCs from wild type (WT) mice and TLR1^-/-^ mice were treated with VCC monomer (10 ng/ml) or pre-formed VCC oligomer (1 μg/ml) for 24 hours. After the incubation, IL-6 production was monitored using ELISA. Buffer and liposome-treated cells served as the negative controls for the experiments with VCC monomer and VCC oligomer, respectively. Data shown here are the averages ± SDs from three independent experiments. *, p < 0.05; **, p < 0.01; one-way ANOVA with Tukey’s multiple comparison test.

Therefore, to examine whether VCC-induced responses in DCs could be mediated via TLR2/6 and/or TLR1/2 engagement, we first performed co-immunoprecipitation-based assays to probe any possible interaction between VCC and TLR2. Interestingly, upon pull-down with anti-VCC antibody, we did not observe any prominent co-immunoprecipitation of TLR2 in the BMDCs treated with either VCC monomer (Fig 1A) or pre-formed VCC oligomer (Fig 1B). Reverse co-immunoprecipitation with anti-TLR2 antibody further confirmed absence of any noticeable interaction between VCC and TLR2 in the BMDCs, treated with either VCC monomer (Fig 1C) or pre-formed VCC oligomer (Fig 1D).

Experiments using BMDCs from the TLR2^-/-^ mice further confirmed that TLR2 was not at all involved in the VCC-mediated DC activation. We used BMDCs from wild type and TLR2^-/-^ mice, and treated the cells with VCC monomer or pre-formed VCC oligomer. Following incubation, we analysed the extra-cellular supernatants for the production of pro-inflammatory cytokine, IL-6, by ELISA. We did not observe any reduction in the IL-6 production from the BMDCs in the absence of TLR2, in comparison to that observed with wild type BMDCs, in response to the VCC monomer/oligomer treatments (Fig 1E). These data suggest that TLR2 is not involved in the induction of VCC-mediated pro-inflammatory responses in DCs.

Interestingly, TLR1 was found to be co-immunoprecipitated with VCC in BMDCs, upon treatment of the cells with either the monomeric (Fig 1F, 1H) or oligomeric form of VCC (Fig 1G, 1I). Involvement of TLR1 in the VCC-mediated pro-inflammatory responses in BMDCs was further confirmed by the siRNA-based knock-down approach. Following siRNA-mediated knock-down of TLR1 in the BMDCs (Fig 1J, left panel), cells were treated with VCC oligomer. Untransfected BMDCs and scrambled siRNA-transfected BMDCs were also treated with VCC oligomer and used as controls. Following incubation, extra-cellular supernatants were analysed for the IL-6 production by ELISA. We observed a significant decrease in the production of IL-6, in the TLR1 siRNA-transfected cells, in comparison to the scrambled siRNA-transfected cells (Fig 1J, right panel), indicating that TLR1 is definitely involved in the VCC-mediated activation of DCs. Further, we employed BMDCs from the wild type and TLR1^-/-^ mice, and following treatment of these cells with either VCC monomer or oligomer, we analysed the extra-cellular supernatants for the production of pro-inflammatory cytokine IL-6. We observed a significant decrease in the IL-6 production in the TLR1^-/-^ BMDCs, in comparison to that in the wild type BMDCs (Fig 1K). These results confirmed that, though TLR2 is not involved, TLR1 is essential for the activation of DCs by both the monomeric and oligomeric forms of VCC.

### VCC monomer and oligomer induce pro-inflammatory signalling in BMDCs via MyD88

It is well established that the majority of the cell surface and intra-cellular TLRs signal via recruitment of the adaptor molecule MyD88 through their cytoplasmic toll/interleukin-1 receptor (TIR) domain [39]. Only exceptions are TLR3, which recruits TRIF, and TLR4 that recruits both TRIF and MyD88 [40,41]. As described above, we observed that DC activation by VCC was mediated through the involvement of TLR1. However, TLR1 does not have a functional TIR domain, and therefore, it cannot recruit MyD88 for initiating the downstream signalling cascade(s) [42]. Therefore, we wanted to confirm whether the downstream adapter molecule MyD88 was at all involved in the VCC-mediated pro-inflammatory response generation in DCs.

**Fig 2:**
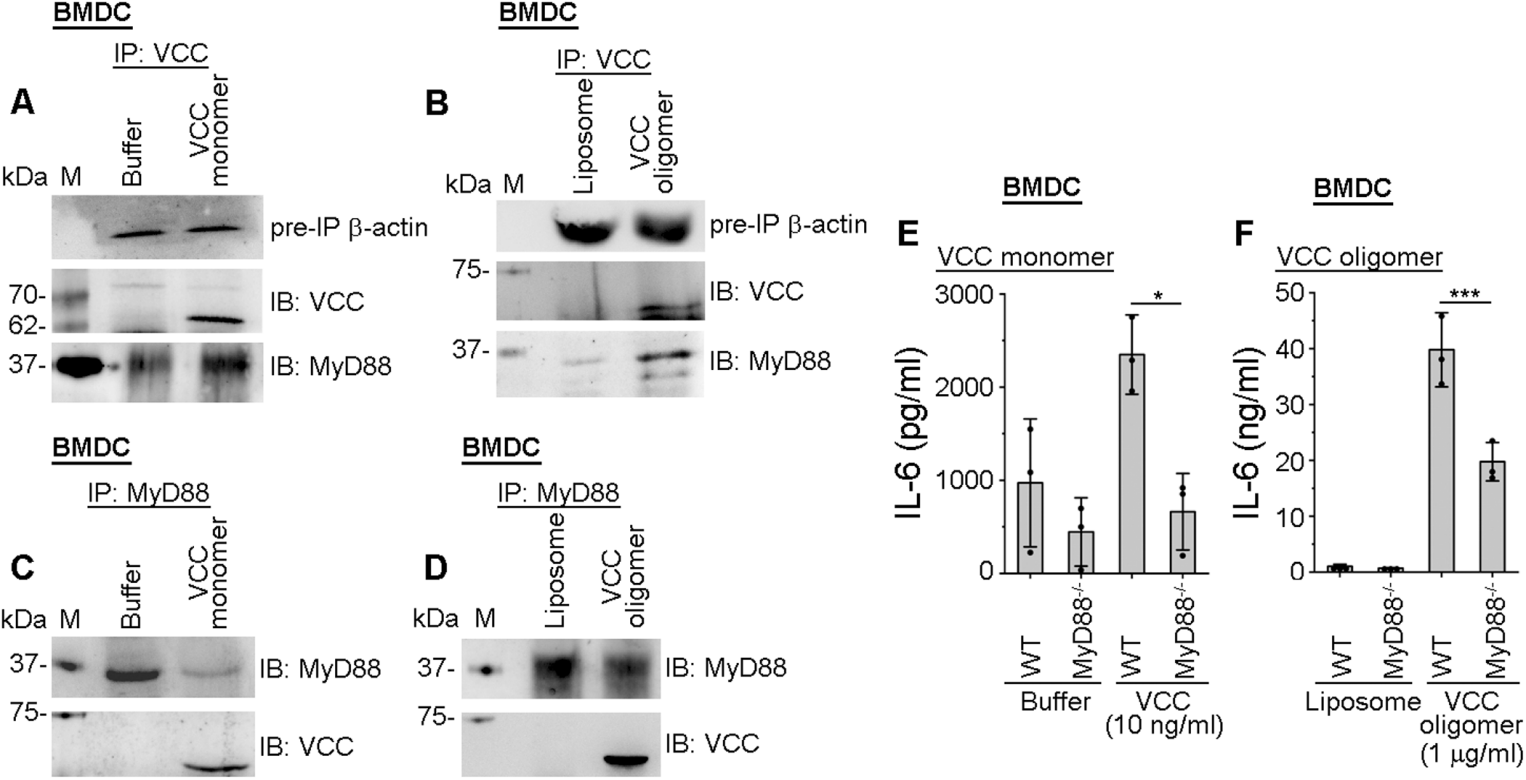
MyD88 is involved in the VCC-induced pro-inflammatory response generation in BMDCs. (A-B) Immunoblot analyses showing co-immunoprecipitation of MyD88 with VCC in BMDCs, treated with VCC monomer (10 ng/ml; A) or pre-formed VCC oligomer (1 μg/ml; B) for 30 minutes. The whole-cell lysates from the treated cells were subjected to immunoprecipitation (IP) with anti-VCC antibody, and immunoprecipitated fractions were subsequently probed for MyD88 and VCC by immunoblotting (IB) using anti-MyD88 and anti-VCC antibodies, respectively. β-actin was probed as the pre-IP protein loading control. Buffer and liposome-treated cells served as the negative controls for the experiments with VCC monomer and oligomer, respectively. Immunoblots shown here are the representatives of three independent experiments. (C-D) VCC co-immunoprecipitated with MyD88 in BMDCs, treated with VCC monomer (10 ng/ml; C) or pre-formed VCC oligomer (1 μg/ml; D) for 30 minutes. The whole-cell lysates from the treated cells were subjected to immunoprecipitation (IP) with anti-MyD88 antibody, and immunoprecipitated fractions were subsequently probed for MyD88 and VCC by immunoblotting (IB) using anti-MyD88 and anti-VCC antibodies, respectively. MyD88 was probed as the protein loading control. Buffer and liposome-treated cells served as the negative controls for the experiments with VCC monomer and oligomer, respectively. Immunoblots shown here are the representatives of three independent experiments. (E-F) Significant reduction in IL-6 production in the VCC-treated BMDCs from MyD88^-/-^ mice, as compared to those from the wild type (WT) mice. BMDCs from the wild type (WT) mice and MyD88^-/-^ mice were stimulated with 10 ng/ml VCC monomer (E) or 1 μg/ml pre-formed VCC oligomer (F) for 24 hours. After the treatment, supernatants were collected and IL-6 production was estimated by ELISA. Buffer or liposome-treated cells served as the negative controls for the experiments with VCC monomer and oligomer, respectively. Data shown here are the averages ± SDs from three independent experiments. *, p < 0.05; ***, p < 0.001; one-way ANOVA with Tukey’s multiple comparison test.

In the co-immunoprecipitation-based assays, we observed that MyD88 co-immunoprecipitated with VCC, upon treatment of the BMDCs with either VCC monomer (Fig 2A, 2C) or pre-formed VCC oligomer (Fig 2B, 2D). These observations suggested that both the monomeric and oligomeric forms of VCC could engage MyD88 in DCs. To further confirm the involvement of MyD88, we employed BMDCs from the MyD88^-/-^ mice. We observed a significant decrease in the IL-6 production in the MyD88^-/-^ BMDCs, in comparison to that in the wild type BMDCs, upon treatment with VCC monomer (Fig 2E), as well as VCC oligomer (Fig 2F). Altogether, our data showed that both VCC monomer and oligomer induced signalling in a MyD88-dependent manner for the DC activation (Fig 2A-2F). This finding, in turn, revealed that TLR-MyD88-based signalling was involved in the VCC-mediated pro-inflammatory response generation in the DCs.

### TLR4 is involved in the VCC-induced pro-inflammatory responses in BMDCs

Though TLR1 and TLR6 can act as the receptors for the PAMPs, they cannot initiate the cascade of pro-inflammatory signalling events on their own, as both of them lack the functional TIR domain, and thus cannot recruit MyD88 through the cytoplasmic TIR domain [42]. Hence, both TLR1 and TLR6 form heterodimers with TLR2 for this purpose. Crucial involvement of MyD88 in the VCC-mediated pro-inflammatory responses in BMDCs, as described above (Fig 2), suggested possible participation of the TLR(s) as the PRR(s) for the initiation of the VCC-mediated signalling in these cells. However, though TLR1 was found to be involved, TLR2 was not essential in the VCC-mediated pro-inflammatory signalling in BMDCs (Fig 1). These data indicated that some other TLR, containing the functional TIR domain, could be heterodimerizing with TLR1 for the induction of the VCC-mediated signalling in DCs.

In an earlier study with VCC monomer, an increase in the surface expression of TLR4, along with TLR2 and TLR6, have been shown in the VCC-treated macrophages [22]. To find out what could be the heterodimerizing partner of TLR1 in the BMDCs, we investigated whether TLR4 could be involved in the recognition of VCC in these cells.

**Fig 3:**
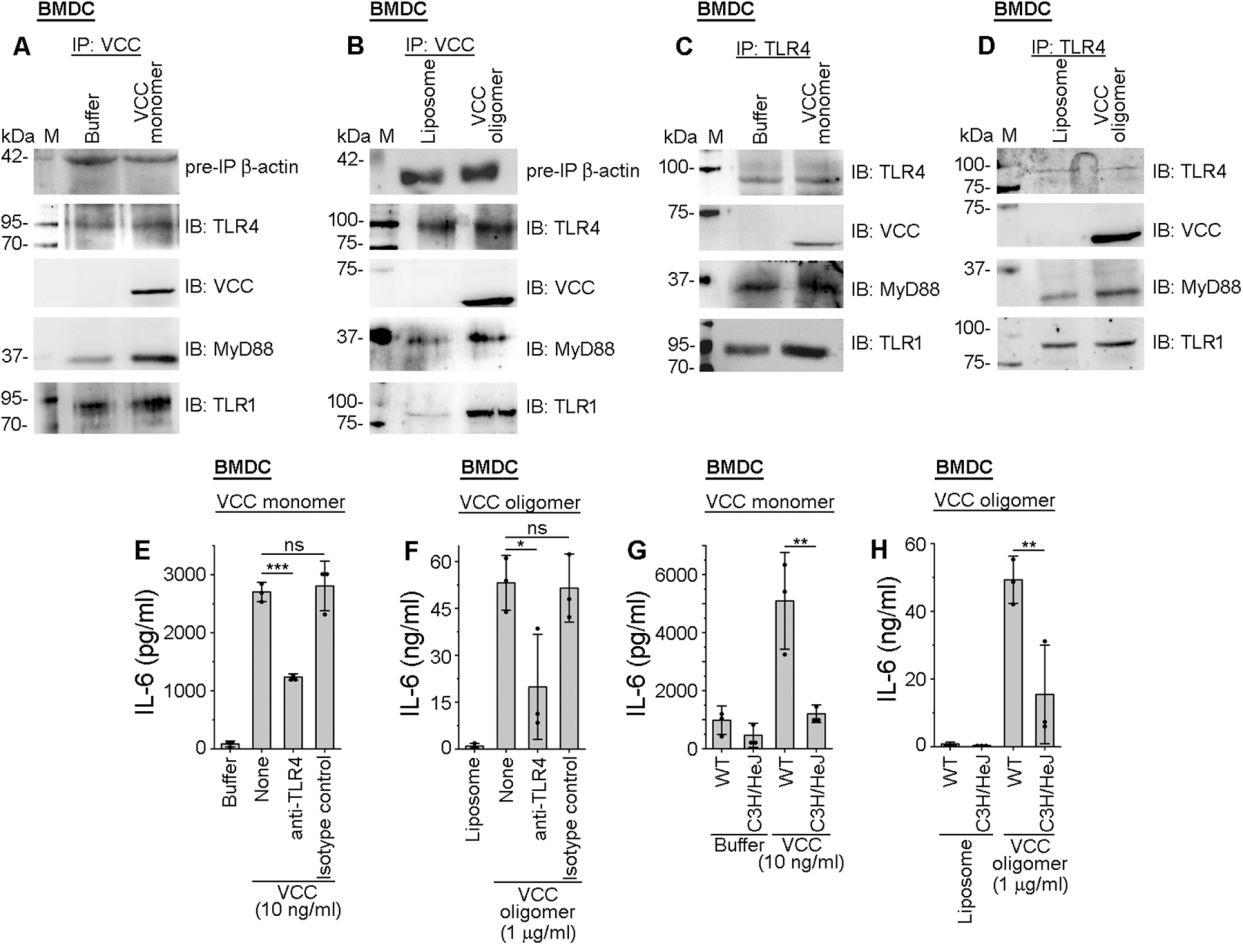
TLR4 is involved in the VCC-induced activation of BMDCs. (A-B) Immunoblot analyses showing association of VCC with TLR4 along with MyD88 and TLR1 in BMDCs, treated with VCC monomer and pre-formed VCC oligomer. BMDCs were treated with 10 ng/ml VCC monomer (A) and 1 μg/ml pre-formed VCC oligomer (B) for 30 minutes. The whole-cell lysates from the treated cells were subjected to immunoprecipitation (IP) with anti-VCC antibody, and the immunoprecipitated fractions were probed by immunoblotting (IB) with anti-VCC, anti-TLR4, anti-TLR1, and anti-MyD88 antibodies. β-actin was probed as the pre-IP protein loading control. Buffer and liposome-treated cells served as controls for the experiments with VCC monomer and oligomer, respectively. Immunoblots shown are the representatives of three independent experiments. (C-D) VCC, MyD88, and TLR1 co-immunoprecipitated with TLR4 in the BMDCs, upon treatment with VCC monomer and pre-formed VCC oligomer. BMDCs were treated with 10 ng/ml VCC monomer (C) or 1 μg/ml pre-formed VCC oligomer (D) for 30 minutes. The whole-cell lysates from the treated cells were subjected to immunoprecipitation (IP) with anti-TLR4 antibody, and the immunoprecipitated fractions were probed by immunoblotting (IB) using anti-VCC, anti-TLR4, anti-TLR1, and anti-MyD88 antibodies. TLR4 served as the protein loading control. Buffer and liposome-treated cells served as the controls for the experiments with VCC monomer and oligomer, respectively. Immunoblots shown here are the representatives of three independent experiments. (E-F) Significant decrease in the VCC-mediated IL-6 production in BMDCs, upon pre-neutralization of TLR4 with anti-TLR4 antibody. BMDCs were pre-treated with anti-TLR4 neutralizing antibody for 2 hours. Following incubation, cells were treated with 10 ng/ml VCC monomer (E) or 1 μg/ml pre-formed VCC oligomer (F), and incubated for 24 hours. After the treatments, IL-6 levels in the extra-cellular supernatants were monitored by ELISA. Cells pre-treated with isotype antibody served as the control for the neutralizing antibody. Buffer and liposome-treated cells served as the negative controls for the experiments with VCC monomer and oligomer, respectively. Data shown here are the averages ± SDs from three independent experiments. ns, non-significant; *, p < 0.05; ***, p < 0.001; one-way ANOVA with Tukey’s multiple comparison test. (G-H) Significant decrease in the VCC-mediated IL-6 production in the BMDCs from the C3H/HeJ mice with abrogated TLR4 signalling, compared to that in the BMDCs from the wild type (WT) mice. BMDCs from the wild type (WT) and C3H/HeJ mice were treated with 10 ng/ml VCC monomer (G) or 1 μg/ml pre-formed VCC oligomer (H) for 24 hours. Following incubation, supernatants were collected and IL-6 production was estimated by ELISA. Buffer and liposome-treated cells served as the negative controls for the experiments with VCC monomer and oligomer, respectively. Data shown here are the averages ± SDs from three independent experiments. **, p < 0.01; one-way ANOVA with Tukey’s multiple comparison test.

Using a pull-down-based assay with anti-VCC antibody, we observed that TLR4 co-immunoprecipitated with VCC along with MyD88 and TLR1 in both VCC monomer- (Fig 3A) and oligomer- (Fig 3B) treated BMDCs. Further, in a reverse-pull-down assay with anti-TLR4 antibody, we observed that TLR4 co-immunoprecipitated with VCC, TLR1 and MyD88, suggesting that TLR4 could be the heterodimerizing partner of TLR1 in response to VCC in the BMDCs (Fig 3C-3D).

To check whether VCC can mediate inflammatory signal via TLR4 in BMDCs, we used TLR4 neutralizing antibody. Upon neutralization of TLR4 in the BMDCs, we observed a significant decrease in the IL-6 production, following treatment with both VCC monomer (Fig 3E) and oligomer (Fig 3F), as compared to that in the isotype antibody-pre-treated (control), and VCC-treated cells (Fig 3E-3F). Further, to confirm the role of TLR4, we used BMDCs from the C3H/HeJ mice. C3H/HeJ mice have a missense mutation in the BB loop of the TIR domain of the TLR4 receptor [43]. We treated BMDCs from both the C3H/HeJ mice and wild type mice with either VCC monomer or oligomer. Following the incubation, extra-cellular supernatants were analysed for the production of pro-inflammatory cytokine, IL-6. We observed a significant decrease in the IL-6 production in the BMDCs from C3H/HeJ mice, compared to that in the cells from wild type mice, upon treatment with both VCC monomer (Fig 3G) and oligomer (Fig 3H).

### VCC induces heterodimerization of TLR1 and TLR4 for the induction of pro-inflammatory signal

**Fig 4:**
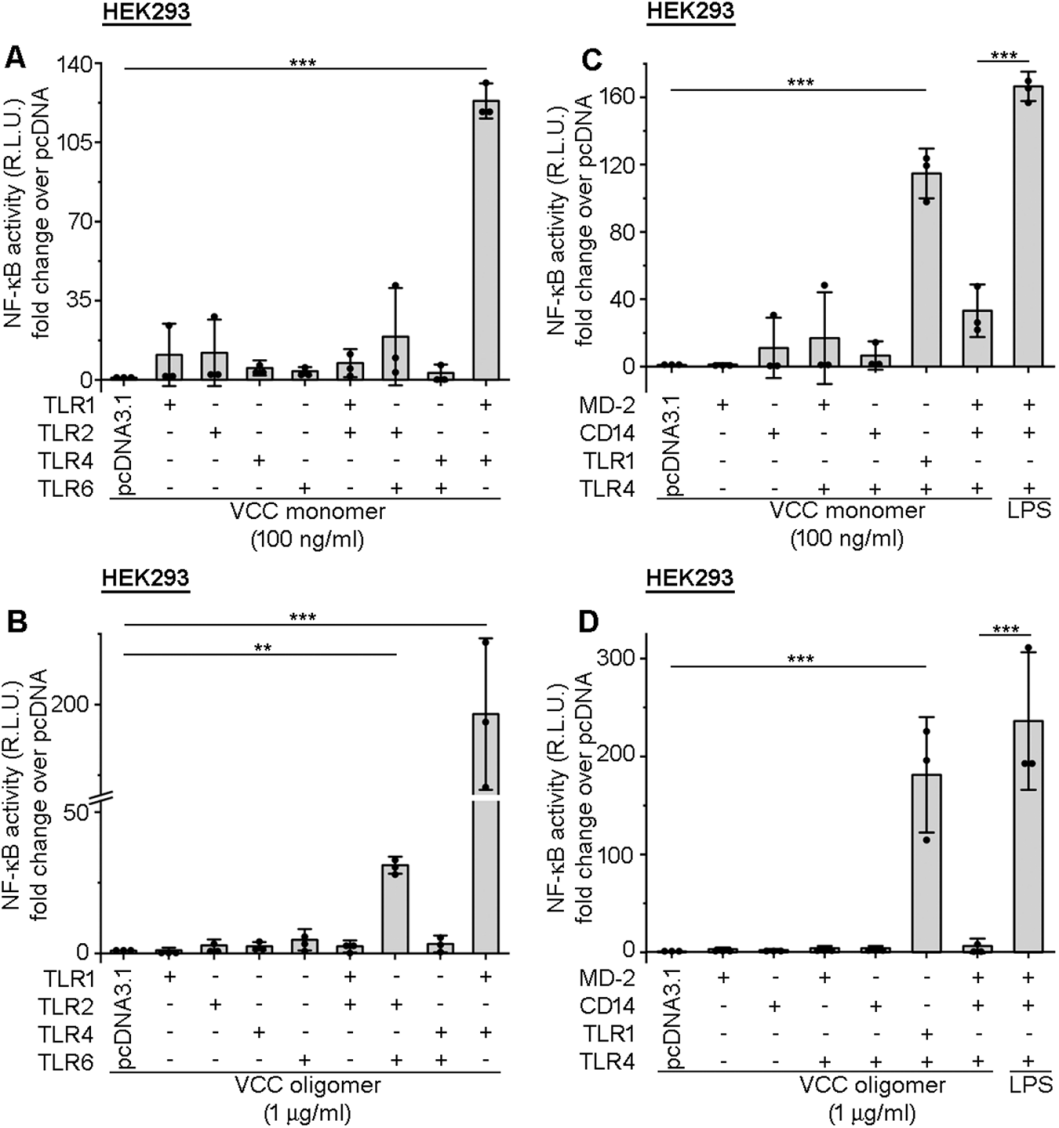
Recombinant co-expression of TLR1-TLR4 allows NF-κB activation in HEK293 cells in response to VCC, while co-expression of the LPS signaling complex (MD-2/CD14/TLR4) does not trigger such response. (A-B) HEK293 cells were transfected with NF-κB Luciferase reporter plasmid, pRL plasmid, along with pcDNA3.1(+)-TLR1, pcDNA3.1(+)-TLR2, pcDNA3.1(+)-TLR4, pcDNA3.1(+)-TLR6, and empty pcDNA3.1(+), in different combinations. After 24 hours, cells were stimulated with 100 ng/ml VCC monomer (A) or 1 μg/ml pre-formed VCC oligomer (B) for additional 12 hours. Empty pcDNA3.1(+)-transfected, VCC-treated cells served as the controls. The NF-κB activation was measured using the Luciferase Reporter Assay. The reporter activity (Relative Luminescence Unit (R.L.U)) was presented in terms of fold change over that in the pcDNA3.1(+)-transfected, VCC-treated cells. Data shown here are the averages ± SDs from three independent experiments. **, p < 0.01; ***, p < 0.001; one-way ANOVA with Dunnett’s multiple comparison test. (C-D) HEK293 cells were transfected with NF-κB Luciferase reporter plasmid, pRL (renilla) plasmid, along with pcDNA3.1(+)-MD-2, pcDNA3.1(+)-CD14, pcDNA3.1(+)-TLR4, pcDNA3.1(+)-TLR1, and empty pcDNA3.1(+), in different combinations. After 24 hours, cells were treated with 100 ng/ml VCC monomer (C), 1 μg/ml pre-formed VCC oligomer (D), or 200 ng/ml LPS (C-D), and the NF-κB reporter activity was measured after 12 hours of the treatments, as described above (in A-B). Empty pcDNA3.1(+)-transfected, VCC-treated cells served as the control. Data shown here are the averages ± SDs from three independent experiments. ***, p < 0.001; one-way ANOVA with Tukey’s multiple comparison test.

To check the specific receptor combination important for the VCC-mediated signalling, we expressed different receptors individually, or in different combinations, in the HEK293 cells. For the expression studies, we cloned different receptors in the pcDNA3.1(+) mammalian expression vector, transfected the recombinant constructs in the HEK293 cells, and checked for their surface expression (S2A-S2E Fig). Transcriptional activation of NF-κB is a major consequence of the TLR-MyD88 signalling. Therefore, we used the cell-based NF-κB Luciferase reporter assay for monitoring the NF-κB activation upon VCC stimulation, to determine the receptor(s) required for the VCC-induced signalling. Cells were transfected with recombinant pcDNA3.1(+) vector harbouring the nucleotide sequence encoding TLR1, TLR2, TLR4, or TLR6 receptors individually, or co-transfected in different combinations, such as TLR1-TLR2, TLR2-TLR6, TLR4-TLR6 and TLR1-TLR4. Subsequently, the transfected cells were stimulated with VCC monomer or pre-formed VCC oligomer. We observed that the expression of TLR1-TLR4 combination in the HEK293 cells could lead to a significant induction of the NF-κB activity, upon treatment with VCC monomer (Fig 4A) and pre-formed VCC oligomer (Fig 4B), suggesting VCC-mediated signalling via TLR1/4 heterodimer. Our data further indicated that TLR4 individually could not induce NF-κB activation signalling in response to either VCC monomer or oligomer in the HEK293 cells, and it needed TLR1 co-expression (Fig 4A-4B), suggesting that the heterodimerization of TLR1/4 was necessary for the VCC-mediated signalling. We also observed that the VCC-induced NF-κB activation via TLR1/4 in HEK293 over-expression system increased in a dose-dependent manner (when tested at the protein concentrations of 5 ng/ml, 10 ng/ml, and 100 ng/ml protein concentrations). However, further increase in the concentration of VCC monomer to 200 ng/ml showed a significant reduction in the NF-κB activation level, presumably due to high cytotoxicity (S2F Fig).

### MD-2 and CD14 do not play any role in VCC recognition and TLR1/4 heterodimerization

Myeloid differentiation factor-2 (MD-2) and CD14 play an important role in the recognition of LPS, and TLR4 signalling [44–46]. We wanted to check if similar to the scenario in the presence of LPS, TLR4 alone, in the presence of CD14 and MD-2, could induce VCC-mediated activation. Further, to eliminate the possibility of TLR4 activation due to LPS contamination in the VCC preparations, if any, we examined the role of CD14 and MD-2, along with TLR4, in the VCC-mediated signalling. Towards that, we co-transfected HEK293 cells with recombinant pcDNA3.1(+) vector harbouring the nucleotide sequence encoding MD-2, CD14, or TLR4 (that are the essential factors for the LPS binding/recognition by the cells), either in isolation or in different combination, as described in the previous section. We observed that stimulation with VCC monomer and oligomer did not lead to any significant NF-κB activation in the HEK293 cells, when transfected with the vectors allowing expression of either CD14, or MD-2, or both, or when co-transfected along with TLR4 (Fig 4C-4D). In contrast, LPS treatment, as the positive control, activated the HEK293 cells leading to the significant NF-κB activation, when co-transfected to express MD-2, CD14, and TLR4 (Fig 4C-4D). It has been reported earlier that, in the endothelial cells, TLR1, through its extracellular domain, abrogates the LPS-induced TLR4 signalling, when overexpressed [47]. This inhibition majorly occurs due to the formation of inactive heterocomplex between TLR1 and TLR4, thus interfering with the ligand binding capacity of TLR4. To check whether VCC can mask the LPS signalling through an increase in the TLR1 surface expression, we assessed the surface expression of TLR1 following TLR1 over-expression in HEK293 cells and subsequent VCC or LPS treatments. We observed an increase in TLR1 surface expression following VCC treatment, and not much change was observed upon LPS treatment (S3A-S3B Fig). Further, to understand whether LPS-mediated signalling could be abrogated upon TLR1 over-expression, we transfected HEK293 cells with either TLR4-MD-2-CD14 or TLR1-TLR4-MD-2-CD14, and treated them with LPS. The NF-κB luciferase assay indicated similar levels of NF-κB activation, suggesting that TLR1 overexpression did not interfere with the TLR4-mediated signalling through LPS, in contrast to that reported earlier [47] (S3C Fig). This contrasting observation led us to question if the TLR1/4 heterodimer formation in the presence of VCC could interfere with the LPS-mediated TLR4 activation. Towards this, we co-transfected HEK293 cells with TLR1, TLR4, CD14 and MD-2, and subsequently treated these cells either with both LPS and VCC simultaneously, or pre-treated them with VCC for two hours followed by LPS treatment. We observed that, in both the cases, there was an attenuation of the NF-κB activation, when compared to only LPS treatment; however, there was a slight increase (although not significant) in the VCC pre-treated, LPS-treated scenario, compared to the VCC and LPS co-treatment (S3D Fig). These observations can be very well explained by the cell tolerance mechanism, as reported in the case of the endotoxin tolerance [48, 49]. Probably, the slight increase in the NF-κB activation in the case of VCC pre-treatment followed by LPS treatment scenario, as compared to the co-treatment together with VCC and LPS (S3D Fig), was due to the fact that VCC had a window of approximately two hours to induce some amount of activation before the presence of LPS could initiate the tolerance mechanism.

Overall, we observed that TLR4 co-expression, only with TLR1, could lead to the VCC monomer and oligomer-induced NF-κB activation. Further, VCC did not require the help of MD-2 and CD14, as it is otherwise observed in the case of LPS. Interestingly, we found that the presence of VCC and LPS together could attenuate the response that was otherwise triggered by LPS alone, probably due to the induction of the tolerance mechanism.

### CD36 is involved in the VCC-induced pro-inflammatory response generation in the BMDCs

Our results so far suggested that both VCC monomer and oligomer induced an unusual heterodimer formation between TLR1 and TLR4. Apart from the commonly known heterodimers between TLR1/2 and TLR2/6, unusual heterodimer formation between TLR4 and TLR6 has been reported in response to the endogenous ligands oxLDL and amyloid-β in the context of sterile inflammation [50]. Such heterodimer formation has been shown to be the outcome of the signalling activation via a known cell-surface co-receptor CD36. CD36 has a large extracellular domain, which binds to a diverse range of ligands. It has hydrophobic pockets that bind to the lipids and lipid-like molecules, and a positively charged lysine cluster, which can bind to the negatively charged DAMPs/PAMPs [51]. CD36 has also been documented previously to act as a TLR co-receptor for many pathogenic and sterile ligands [52–55]. Therefore, we also wanted to assess the role of CD36 in the VCC-mediated responses through the activation of TLR1/4. Towards this direction, we first examined whether CD36 was involved in the VCC-mediated activation of the BMDCs. In the immunoprecipitation-based assays using anti-VCC antibody, we observed prominent CD36 co-immunoprecipitation with VCC in the BMDCs, treated with both VCC monomer (S4A Fig), and pre-formed VCC oligomer (S4B Fig). Further, with reverse co-immunoprecipitation with anti-CD36 antibody, we confirmed that both the forms of VCC could associate with CD36 (S4C-S4D Fig). For further confirmation of the involvement of CD36 in the VCC-induced pro-inflammatory responses, we treated CD36^-/-^ and wild type BMDCs with VCC monomer and oligomer, and observed a significant decrease in the IL-6 production in the absence of CD36 in BMDCs (S4E-S4F Fig). These data suggested that CD36 played an important role in the VCC-mediated pro-inflammatory response generation in DCs.

### VCC-mediated TLR1/TLR4 heterodimerization is probably independent of CD36

To check whether TLR1/TLR4 heterodimerization required help from CD36, we transfected HEK293 cells with recombinant pcDNA3.1(+) vector harbouring the nucleotide sequence encoding TLR1, TLR4 or CD36, individually, or in different combinations (S2 Fig). Using NF-κB reporter assay, we observed that TLR1 and TLR4 co-expression in the HEK293 cells could trigger a significant amount of NF-κB activation in response to both VCC monomer and oligomer (Figs 4, S4G, and S4H). However, we also observed that the recombinant expression of CD36 alone in HEK293 cells could induce significant NF-κB activation upon VCC stimulation (S4G-S4H Fig), suggesting that CD36 itself could possibly act as a pro-inflammatory receptor or PRR, for both VCC monomer and oligomer, and probably not as a co-receptor (S4G- S4H Fig). Further we observed a higher extent of NF-κB activation in the HEK293 cells, co-expressing TLR1, TLR4, and CD36 together, in comparison to those in the HEK293 cells, either co-expressing TLR1 and TLR4, or expressing CD36 alone, upon stimulation with VCC monomer or oligomer. These observations suggested towards a possible additive effect of CD36 along with TLR1/TLR4 heterodimer in generating the pro-inflammatory responses upon VCC stimulation. Our data indicated that, though CD36 might not be necessary as a co-receptor for the TLR1/TLR4 heterodimerization, however, CD36 along with TLR1/TLR4 heterodimer could be involved in augmenting the VCC-mediated pro-inflammatory response generation.

### VCC engages with both TLR2 and TLR4 in the macrophages for the production of pro-inflammatory cytokines

**Fig 5:**
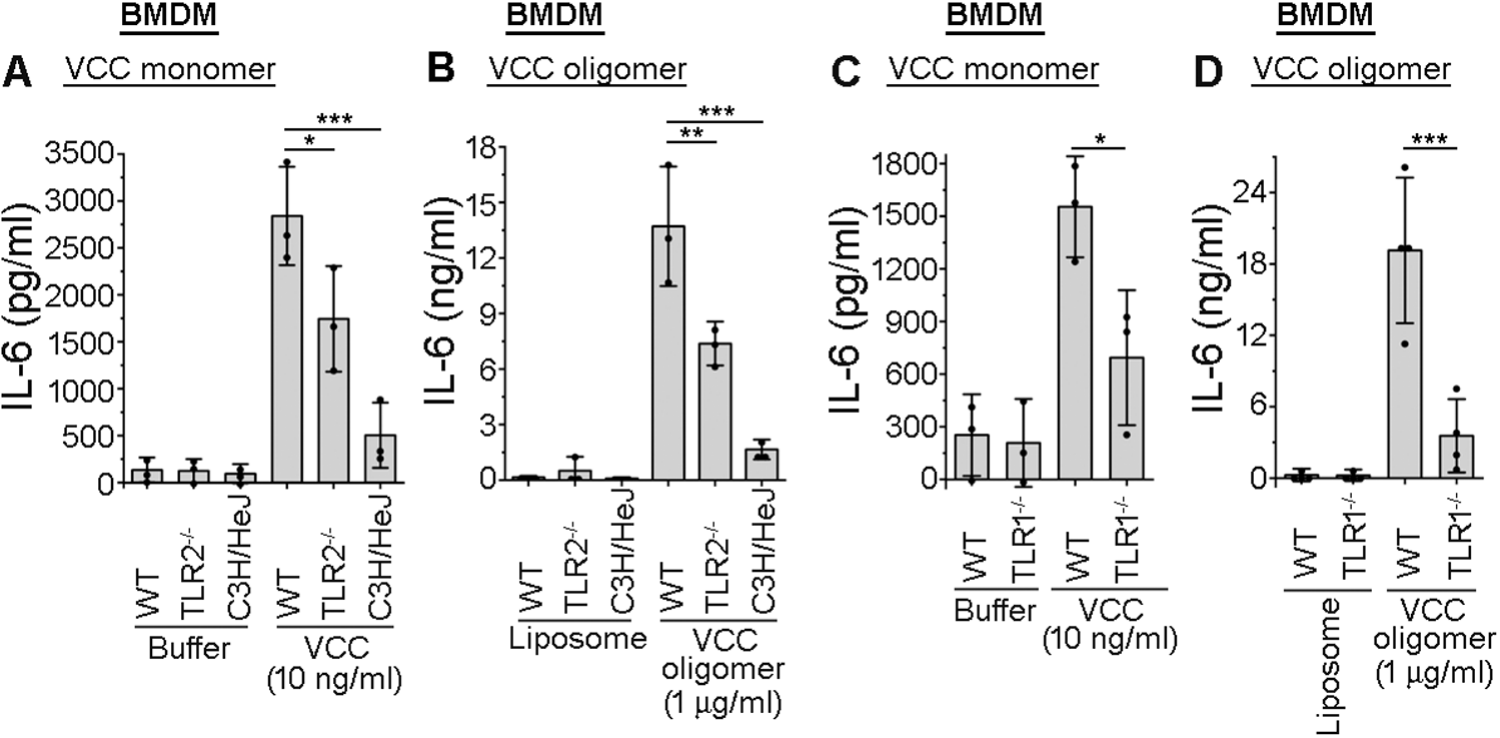
VCC-induced pro-inflammatory response generation in BMDMs is dependent on TLR2, TLR4 as well as TLR1. Significant decrease in the VCC-mediated IL-6 production in BMDMs from TLR2^-/-^ (A-B), C3H/HeJ (A-B), and TLR1^-/-^ (C-D) mice, as compared to that in BMDMs from the wild type (WT) mice. BMDMs from wild type (WT), TLR2^-/-^, C3H/HeJ, and TLR1^-/-^ mice were treated with 10 ng/ml VCC monomer (A, C) and 1 μg/ml VCC oligomer (B, D). After 24 hours of treatment, the supernatants were analysed for the IL-6 production using ELISA. Buffer and liposome-treated cells served as negative controls for the experiment with VCC monomer and oligomer, respectively. Data shown here are the averages ± SDs from three to four independent experiments. *, p < 0.05; **, p < 0.01; ***, p < 0.001; one-way ANOVA with Tukey’s multiple comparison test.

It has been observed in a previous study that VCC oligomer can induce activation of the macrophages via TLR2 [10]. However, in the present study, we observed that VCC oligomer induced activation of DCs via TLR1 and TLR4 (Figs 1 and 3). These apparently contradicting observations prompted us to examine whether TLR4 could also be important in the VCC-mediated macrophage activation. Towards this direction, we used bone marrow-derived macrophages (BMDMs) from wild type, TLR2^-/-^ and C3H/HeJ (having abortive TLR4 function) mice, and treated the cells with VCC monomer or pre-formed VCC oligomer. Following the incubations, extracellular supernatants were analysed for the production of IL-6 by ELISA. We observed that in the absence of either TLR2 or TLR4 functioning, there was significant decrease in the IL-6 production by the BMDMs, in response to VCC monomer and oligomer (Fig 5A-5B). As TLR1/4 heterodimer was involved in the VCC-mediated activation of DCs, we wanted to check whether along with TLR4, TLR1 could also be involved in the VCC-mediated activation of macrophages. We used BMDMs from the TLR1^-/-^ mice, and observed a significant decrease in the IL-6 production, in comparison to that in the BMDMs from the wild type mice, in response to both VCC monomer and oligomer treatments (Fig 5C-5D). These observations suggested that in the macrophages, VCC engaged both TLR2 and TLR4, and in addition to the TLR2/6 heterodimer [10], it probably also induced pro-inflammatory responses via TLR1/4 heterodimer engagement.

### Differential expressions of TLRs in macrophages and DCs are probably responsible for the engagement of different receptor combinations in response to VCC

Our data so far revealed that in the DCs, VCC monomer- and oligomer-mediated pro-inflammatory signals were generated preferably through the TLR1/4 heterodimer. In contrast in the macrophages, both TLR2/6 and TLR1/4 were found to be the mediators of the pro-inflammatory signals in response to VCC. Further, in the TLR co-transfection assay in the heterologous HEK293 cell system, we observed that TLR2 and TLR6 co-expression could also induce NF-κB activation upon stimulation with both VCC monomer and oligomer, though not to the extent observed with the TLR1/4 expression combination (Fig 4A-4B). These observations led us to think that the differential surface expression of the receptors, and thus their different availability for the ligand recognition, in the macrophages and DCs, could be a reason behind the observed involvement of different TLR combinations in the two cell types, during VCC-induced signalling processes. Towards understanding the reason for the differential recognition of the same ligand with different receptor combinations in the BMDCs and BMDMs, we checked the surface expression of the TLRs in both the cell types at the basal level, as well as following the treatments with both VCC monomer and oligomer.

We differentiated both DCs and macrophages from the bone marrow of C57BL/6 mice. We analysed the basal level surface expression of TLR1, TLR2, TLR4 and TLR6 on the differentiated BMDCs and BMDMs, and compared with those in the undifferentiated cells, using flow cytometry. We observed that upon differentiation into BMDCs, even at the basal level, surface expression of TLR1 was ~2-fold and that of TLR4 was more than 3-fold higher compared to the undifferentiated cells, while the surface expression of TLR2 and TLR6 showed ~2.5 and ~1.5-fold increase, respectively, although not statistically significant, in BMDCs compared to the undifferentiated cells (S5A-S5D Fig). In contrast, upon differentiation into the BMDMs, basal level surface expression of TLR2 increased to almost 5 times to that of the undifferentiated cells, expressions of TLR4 and TLR6 increased to ~2.5-fold and ~1.5-fold, respectively, compared to the undifferentiated cells, while the TLR1 surface expression was similar to that of the undifferentiated cells (S5A-S5D Fig). Altogether, these data suggested differential surface availability of different TLRs in the BMDCs and BMDMs, even at the basal level. These results, in turn, provided possible explanation for the different preferred receptor combinations, involved during VCC-mediated pro-inflammatory response generation in the two cell types.

Upon observing differential basal surface expression profiles of the different TLRs in BMDCs and BMDMs, we examined whether VCC treatment could mediate any further change in the surface expressions of these PRRs. For this, BMDCs and BMDMs were treated with VCC monomer and pre-formed VCC oligomer for different time points, and surface expressions of the TLRs were monitored by flow cytometry (S6 and S7 Figs). Buffer- and liposome-treated cells were also analysed as the controls for the experiments with VCC monomer and oligomer, respectively (S6 and S7 Figs).

We found that the treatment of BMDCs with VCC oligomer resulted in a 2 to 4-fold increase (although not statistically significant) in the TLR1 surface expression compared to the liposome-treated control cells, at different time points tested. TLR4 surface expression also showed ~1.5-fold increase (although not statistically significant) compared to liposome-treated BMDCs, at all the time points tested. In contrast, there was no detectable increase in the surface expression of TLR2 and TLR6 as compared to that in the liposome-treated cells at any of the time points (S6A- S6D Fig). In case of VCC monomer treatment in BMDCs, we observed ~1.5-fold increase in the surface expression of TLR4 and TLR1 as compared to the buffer-treated BMDCs. Similar to our observations with VCC oligomer, there was no increase in the surface expression of TLR2 and TLR6, in the VCC monomer-treated BMDCs, compared to those in the buffer-treated cells (S7A- S7D Fig).

Taken together, these results revealed that BMDCs not only showed higher basal-level surface expressions of TLR1 and TLR4 compared to the undifferentiated cells (S5 Fig), surface expressions of these PRRs got augmented further upon exposure to VCC (S6 and S7 Figs). Thus, the increased surface availability of TLR1 and TLR4 in the BMDCs, at the basal level as well as upon VCC encounter, was probably the reason why these two PRRs appeared to be the preferred receptors for VCC (both the monomeric and oligomeric form) for mediating the pro-inflammatory signals in DCs.

Upon treatment of the BMDMs with VCC oligomer, we observed ~1.5-fold increase in the surface expression of TLR2 at all the time points, and a slight increase in the surface expression of TLR6, over the control cells treated with liposomes. However, there was no increase in the TLR1 and TLR4 surface expression upon VCC oligomer treatment, compared to those in the liposome-treated cells (S6A-S6D Fig). Further, upon VCC monomer treatment of the BMDMs, we observed upto 1.5-fold increase in the surface expressions of TLR2 and TLR6, but no change in the surface expressions of TLR1 and TLR4, compared to the buffer-treated cells (S7A-S7D Fig).

Taken together, our results revealed that BMDMs, at the basal level, showed 5-fold, 2.5-fold, and 1.5-fold higher surface expressions of TLR2, TLR4 and TLR6, respectively, in comparison to the undifferentiated cells, while no increase in the TLR1 surface expression level was observed (S5 Fig). Our data further demonstrated that upon exposure to VCC monomer as well as VCC oligomer, BMDMs showed a 1.5-fold increase in the TLR2 as well as TLR6 surface expression, but no detectable increase in the surface expressions of TLR1 and TLR4 (S6 and S7 Figs). Therefore, in the BMDMs, increased surface availability of both TLR2 and TLR6, at the basal level as well as upon VCC stimulation, makes them important receptors for VCC engagement. Further, our data also revealed a 2-fold increase in the TLR4 surface expression in the BMDMs in the basal level upon differentiation (S5 Fig). It is also possible that, even in the undifferentiated bone marrow-derived cells, and thus in the BMDMs, there could be some basal surface expression of TLR1. Accordingly, the basal level availability of TLR1 and TLR4 in the BMDMs, although not very high, still plays some crucial roles in the VCC recognition, as observed in the compromised IL-6 production in the BMDMs that lacked functional TLR1 or TLR4 (Fig 5A-5D). Therefore, based on the surface expression profiles of the different TLRs in the undifferentiated bone marrow-derived cells, and in the differentiated BMDMs (at the basal level and upon VCC treatment), it is possible to conclude that in macrophages, majorly TLR2 and TLR6 are available as the receptors for VCC, while TLR1 and TLR4 are also probably available at the initial time points of the ligand engagement.

### VCC-mediated pro-inflammatory responses *in vivo* involve TLR1, TLR4, and TLR2

**Fig 6:**
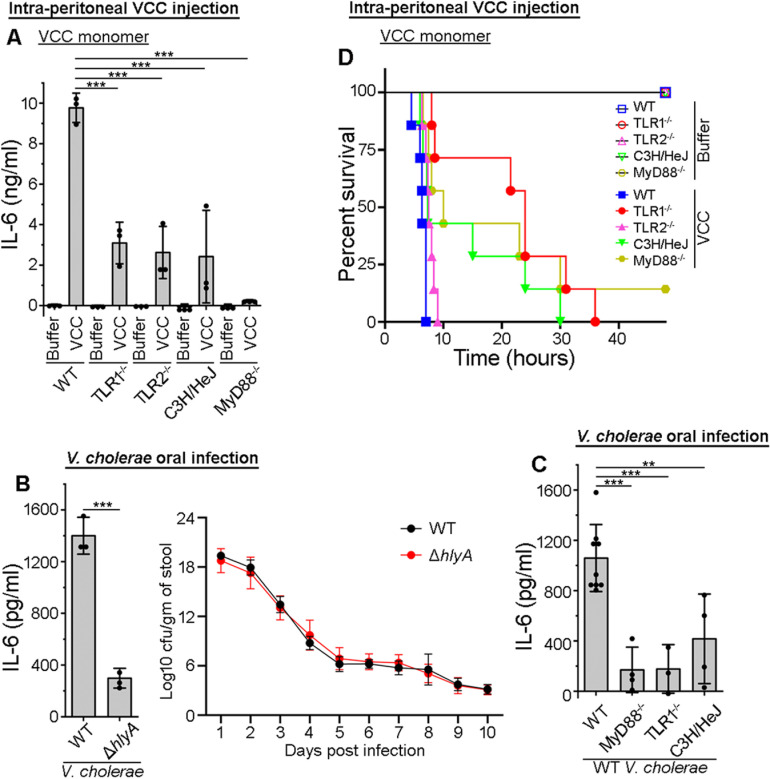
VCC-induced in vivo pro-inflammatory response and toxicity are TLR-dependent. (A) Decrease in serum IL-6 levels in TLR1^-/-^, TLR2^-/-^, C3H/HeJ and MyD88^-/-^ mice, in comparison to that in the wild type (WT) mice upon intra-peritoneal administration of VCC. WT, TLR1^-/-^, TLR2^-/-^, C3H/HeJ and MyD88^-/-^ mice were injected intra-peritoneally with 5 μg VCC (in 100 μl PBS), and blood was collected after 4 hours. Serum was obtained, and IL-6 level in the serum was estimated by ELISA. WT and the transgenic mice injected with equal volume of PBS served as the negative controls. Data shown here are the averages ± SDs from three independent experiments. ***, p < 0.001; one-way ANOVA with Tukey’s multiple comparison test. (B) Left panel shows the decrease in serum IL-6 levels in mice infected with VCC-deleted (Δ*hlyA*) *V. cholerae* variant, in comparison to those in mice infected with the wild type (WT) *V. cholerae*. Right panel shows the colonization efficacies of WT and Δ*hlyA V. cholerae*. Mice were infected orally with the bacteria (5x10^9^ cells). After 4 hours of infection, serum was obtained from the blood, and IL-6 level in the serum was estimated by ELISA (left panel). For the colonization assay, mice stools were collected for 10 consecutive days after the infection, weighed, and plated on the TCBS agar. Line graph represents the Log10 cfu/gram of the stool for 10 days (right panel). Data shown here are the averages ± SDs from three (for the serum IL-6 measurements) to four (for the colonization assay) independent experiments. ***, p < 0.001; Student’s unpaired t-test. (C) Decrease in the serum IL-6 levels in TLR1^-/-^, C3H/HeJ and MyD88^-/-^ mice, in comparison to that in the wild type (WT) mice upon oral infection with wild type (WT) *V. cholerae*. Wild type (WT), TLR1^-/-^, C3H/HeJ and MyD88^-/-^ mice were orally infected with *V. cholerae*, and blood was collected after 4 hours. Serum was obtained, and IL-6 level in the serum was estimated by ELISA. Data shown here are the averages ± SDs from three or more independent experiments. **, p < 0.01; ***, p < 0.001; one-way ANOVA with Dunnett’s multiple comparison test. (D) Increased survival of TLR1^-/-^, C3H/HeJ and MyD88^-/-^ mice, as compared to that of the WT mice, upon VCC intoxication. WT, TLR1^-/-^, TLR2^-/-^, C3H/HeJ and MyD88^-/-^ mice were injected intra-peritoneally with 5 μg VCC (in 100 μl PBS), and the survival of the animals was monitored. Kaplan-Meier plot of cumulative mortality is presented to compare the survival rate. WT and transgenic mice injected with equal volume of PBS only served as the control. For the VCC-treated mice in each group, n=7 (1 mouse/experiment was taken in each group, and the experiment was performed 7 times). For the negative control, 1 mouse/experiment was also taken in each group that was injected with PBS (n=7). However, for the simplified presentation purpose, the survival time-point for one PBS-treated control mouse from each group is shown in the graph.

We observed that TLR1/4 heterodimer played an important role in the VCC-mediated pro-inflammatory responses in DCs and macrophages. Additionally, TLR2 was also found to be involved in the VCC-mediated pro-inflammatory responses in macrophages. To further understand the role(s) of these TLRs in the induction of pro-inflammatory responses *in vivo*, we administered single dose of 5 μg VCC monomer (in 100 μl), intra-peritoneally, in wild type (C57BL/6) and transgenic (TLR1^-/-^, TLR2^-/-^, C3H/HeJ and MyD88^-/-^) mice, and collected the serum after 4 hours of treatment. We observed that the IL-6 production was significantly less in the serum collected from the VCC-treated transgenic mice, in comparison to that in the VCC-treated wild type mice (Fig 6A). These results suggest that the TLR1/TLR4 heterodimer is necessary for the VCC-mediated inflammation. Further, our results imply that TLR2 also plays a crucial role in the induction of VCC-mediated inflammatory condition *in vivo* (Fig 6A).

To examine the effect of VCC under a more physiologically relevant setting of a *V. cholerae* infection scenario *in vivo*, we used wild type and VCC-deficient (Δ*hlyA)* strains of *V. cholerae. V. cholerae* is an enteric pathogen, hence, follows the oral route of infection. Therefore, we orally infected the mice (wild type C57BL/6 mice) with wild type and Δ*hlyA* variants of *V. cholerae* and assessed the pro-inflammatory cytokine levels in the serum. We observed a significant reduction in the IL-6 levels in the serum of mice infected with the Δ*hlyA V. cholerae* as compared to those infected with the wild type bacteria (Fig 6B). However, the absence of VCC did not affect the colonization of the bacteria in the mice gut as observed using the bacterial shedding assay (Fig 6B). This result confirmed the potent role of VCC in the pro-inflammatory response generation during *V. cholerae* infection in mice. Furthermore, through experiments involving mice with wild type (C57BL/6), TLR1^-/-^, C3H/HeJ and MyD88^-/-^ background, we observed that the *V. cholerae*-mediated VCC-induced inflammation *in vivo* was critically dependent on the TLR1/4 and MyD88 signalling pathway (Fig 6C).

### Absence of MyD88, TLR1 and TLR4 imposes considerable delay in the VCC-induced mortality in mice

While monitoring the VCC-mediated pro-inflammatory cytokine production in mice *in vivo*, we observed that the intra-peritoneal/systemic administration of VCC monomer was fatal for the mice. At the selected dose of 5 μg of VCC (in 100 μl), administered intra-peritoneally, we observed a survival window of about 4-7 hours in the wild type mice (Fig 6D). When we checked for the survival of the transgenic, TLR1^-/-^ and C3H/HeJ, mice with the similar dose of VCC monomer, we observed an increased survival, for up to more than 30 hours, suggesting that TLR1 and TLR4 play an important role in the VCC-mediated toxicity in the animals (Fig 6D). However, in the absence of TLR2 (in the TLR2^-/-^ transgenic mice), we observed a similar level of mortality as observed with the wild type mice, thus suggesting that TLR2 plays a role in the pro-inflammatory responses only, but not in the VCC-induced toxicity (Fig 6A and 6D). Notably, we also observed an increased survival in mice in the absence of MyD88 (in the MyD88^-/-^ transgenic mice), thus confirming a crucial role of the TLR1/4-mediated signalling in the VCC-mediated toxicity *in vivo* (Fig 6D).

### VCC is the major toxin involved in the *V. cholerae*-mediated killing of the immune cells

**Fig 7:**
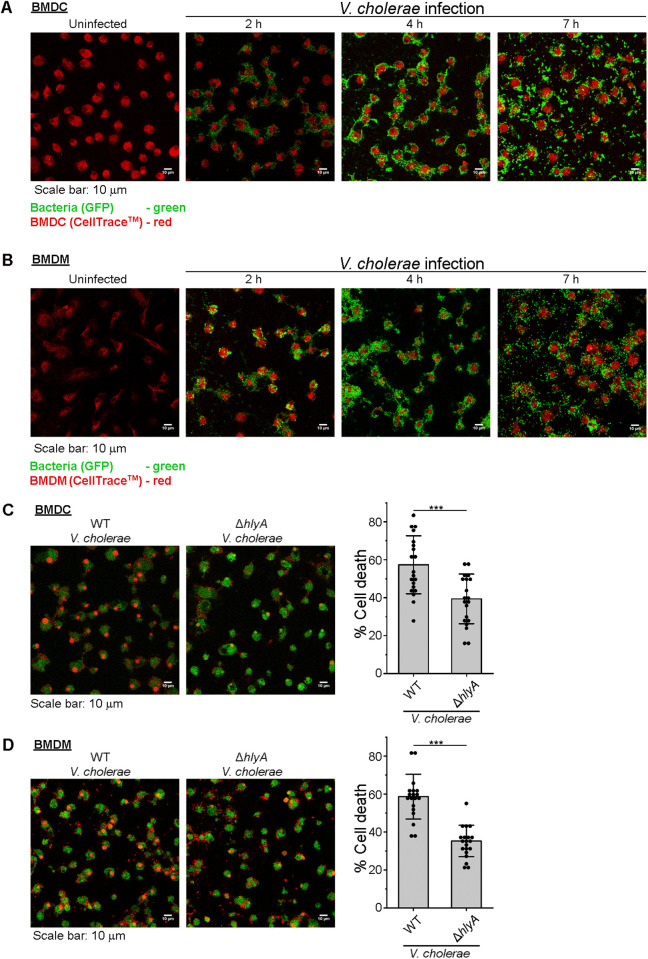
*V. cholerae* forms biofilm on BMDCs and BMDMs leading to VCC-mediated cell death. (A-B) Confocal microscopy images showing *V. cholerae* attachment to the BMDCs and BMDMs, formation of biofilms and dispersal from the immune cell surface. BMDCs (in A) and BMDMs (in B) were infected with *V. cholerae* at a multiplicity of infection (MOI) of 64 for 2, 4 and 7 hours. Uninfected cells served as the controls. Cells were stained with CellTrace (shown in red), and biofilm formation was visualized with the GFP-tagged bacteria (shown in green). A total of 16 to 19 images from three independent experiments were taken for the uninfected, and for each of the time points of the infected cells, and the representative images are shown here. (C-D) Decrease in cell death in BMDCs (shown in C) and BMDMs (shown in D) upon infection with VCC-deleted (Δ*hlyA*) variant of *V. cholerae* in comparison to the cells infected with wild type (WT) bacteria. Confocal microscopy images showing the extent of cell death in BMDCs and BMDMs, upon co-culturing in the presence of WT and Δ*hlyA* variants of *V. cholerae*. BMDCs/BMDMs were co-cultured with the *V. cholerae* variants for 7 hours. Cells were stained with CellTrace (shown in green), and cell death was visualized with propidium iodide (PI) staining (shown in red). A total of 19 to 20 images, from three independent experiments, were taken for each of the WT and Δ*hlyA*-infected cells, and the representative images are shown here. Bar graphs (in the right) show the percentage of cell death in the WT and Δ*hlyA V. cholerae*-infected BMDCs (C) and BMDMs (D). Data shown here are the averages ± SDs. ***, p < 0.001; Student’s unpaired t-test.

It has recently been demonstrated that *V. cholerae* form biofilm surrounding the immune cells, and kill them, thus highlighting an aggressive role of biofilm formation in the bacterial pathogenesis [56]. To investigate whether biofilm formation occurs on the BMDCs and BMDMs upon infection with *V. cholerae*, we infected the cells for different time points. Consistent with the findings reported earlier, we observed that the bacterial cells began to attach to the immune cells after 2 hours of co-culture (Fig 7A-7B). By the 4-hour mark, we noted accumulation of the bacterial cells over the immune cells (Fig 7A-7B), consistent with the observations reported earlier [56]. At the later time point of 7 hours, we observed the dispersal of the bacterial cells from the immune cells (Fig 7A-7B), presumably after the induction of the immune cell death.

We further wanted to probe for the immune cell death following the biofilm formation on the BMDCs and BMDMs, and the role of VCC in such cell killing process. For this, we infected BMDCs and BMDMs with wild type or Δ*hlyA V. cholerae.* For probing the death of the BMDCs and BMDMs, we monitored propidium iodide (PI) incorporation into the nucleus, as PI intercalates into the DNA of the dead or near-dead cells, when the cell membranes are either disintegrated, or become porous enough to allow the entry of PI. We observed prominent accumulation of PI in BMDCs infected with wild type *V. cholerae*, while a significantly lesser PI accumulation was documented for the cells infected with the Δ*hlyA* mutant strain. Thus, these results suggested the importance of VCC in killing the BMDCs, upon co-culturing in the presence of *V. cholerae* (Fig 7C). Wild type *V. cholerae* infection also resulted in the PI accumulation in the BMDMs, and a significant decrease in the PI accumulation in the BMDMs was observed when infected with the Δ*hlyA* mutant (Fig 7D).

### VCC-induced cell death involves TLR-mediated signalling

**Fig 8:**
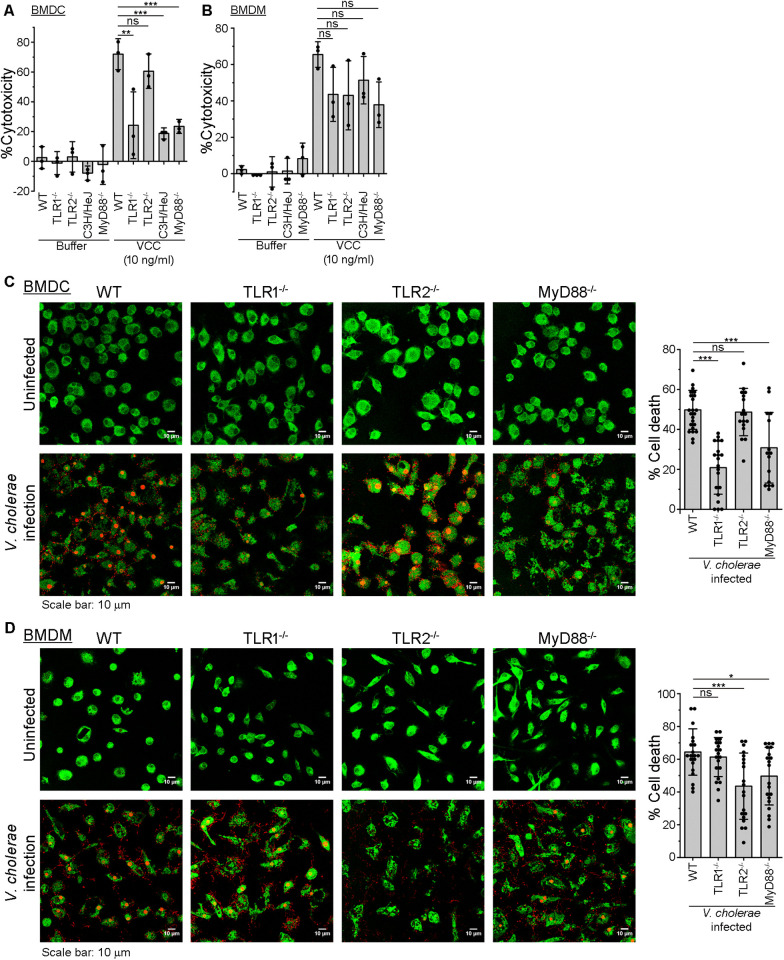
VCC as well as *V. cholerae* induce cell death in BMDCs and BMDMs in a TLR-dependent manner. (A-B) LDH-release assay of cytotoxicity showing the extent of VCC-induced cell death in BMDCs (A) and BMDMs (B) from wild type (WT), TLR1^-/-^, TLR2^-/-^, C3H/HeJ, and MyD88^-/-^ mice. BMDCs/BMDMs from WT and transgenic mice were treated with 10 ng/ml VCC monomer for 12 hours. Following treatments, supernatants were collected, and %cytotoxicity was measured by LDH-release assay. Buffer-treated cells served as the negative controls. Data shown here are the averages ± SDs of %cytotoxicity from three independent treatments. ns, non-significant; **, p < 0.01; ***, p < 0.001; one-way ANOVA with Tukey’s multiple comparison test. (C-D) Confocal microscopy images showing the extent of cell death in BMDCs (C) and BMDMs (D) from wild type (WT), TLR1^-/-^, TLR2^-/-^ and MyD88^-/-^ mice, upon co-culturing in the presence of *V. cholerae* (lower panels in C and D). BMDCs/BMDMs were co-cultured with *V. cholerae* for 7 hours. Uninfected cells served as the controls (upper panels in C and D). Cells were stained with CellTrace (green), and cell death was visualized with propidium iodide (PI) staining (red). A total of 15 to 20 images, from three (for BMDCs) to four (for BMDMs) independent experiments, were taken for each of the uninfected and infected cells from the WT, TLR1^-/-^, TLR2^-/-^ and MyD88^-/-^ mice, and the representative images are shown here. Bar graphs (in the right) show the percentage of cell death in the *V. cholerae*-infected BMDCs (in C) and BMDMs (in D) from the WT, TLR1^-/-^, TLR2^-/-^ and MyD88^-/-^ mice. Data shown here are the averages ± SDs. ns, non-significant; *, p<0.05; ***, p < 0.001; one-way ANOVA with Dunnett’s multiple comparison test.

As described above, we observed increased survival of mice in the absence of TLR1, TLR4 and MyD88, but not in the absence of TLR2. Based on such observation, we wanted to check whether TLR1/4-mediated signalling could play any role in killing the target cells upon VCC-treatment. For this, we treated BMDCs from wild type and transgenic (MyD88^-/-^, TLR1^-/-^, TLR2^-/-^, and C3H/HeJ) mice with VCC monomer. Result of the LDH-release assay showed a significant reduction in cytotoxicity in BMDCs lacking MyD88, TLR1 and TLR4, as compared to that in BMDCs from the wild type mice. This result indicated a prominent involvement of TLR1 and TLR4, and a possible role of TLR1/TLR4/MyD88-mediated signalling axis in the VCC-induced cell death in BMDCs (Fig 8A). Additionally, we observed similar level of cytotoxicity in BMDCs from the TLR2^-/-^ and wild type mice in the presence of VCC (Fig 8A). Altogether, these observations suggest that in DCs the VCC-induced cytotoxicity is specifically dependent on TLR1/4, and TLR2 does not play any role (Fig 8A).

Further, we checked whether TLRs also play any role in the VCC-induced cytotoxicity in macrophages. Although not statistically significant, we observed some noticeable decrease in the VCC-mediated cytotoxicity in the BMDMs from MyD88^-/-^, TLR1^-/-^, C3H/HeJ and TLR2^-/-^ mice, in comparison to that in the BMDMs from the wild type background (Fig 8B).

### TLR-mediated signalling plays an important role in the death of the immune cells such as DCs and macrophages following *V. cholerae* infection

In a very important recent study, it has been demonstrated that *V. cholerae* forms biofilm surrounding the immune cells, and then kill them by secreting VCC [56]. To check whether the TLR(s) have any role to play during this immune cell killing following *V. cholerae* infection, we infected BMDCs and BMDMs from wild type as well as transgenic (MyD88^-/-^, TLR1^-/-^, TLR2^-/-^) mice with *V. cholerae*. We observed prominent accumulation of PI in the wild type and TLR2^-/-^ BMDCs, while noticeably less PI accumulation was observed in the cases of TLR1^-/-^ and MyD88^-/-^ BMDCs, suggesting that TLR1 and MyD88 are crucial for the *V. cholerae*-mediated killing of the BMDCs (Fig 8C). Interestingly, such responses were not found to be affected, at least to any noticeable extent, in the BMDMs from the TLR1^-/-^ transgenic mice. However, significant reduction was observed in the BMDMs from the TLR2^-/-^ and MyD88^-/-^ transgenic mice, in comparison to that in the wild type BMDMs (Fig 8D).

### VCC induces activation of the neutrophils in a TLR-dependent manner

**Fig 9:**
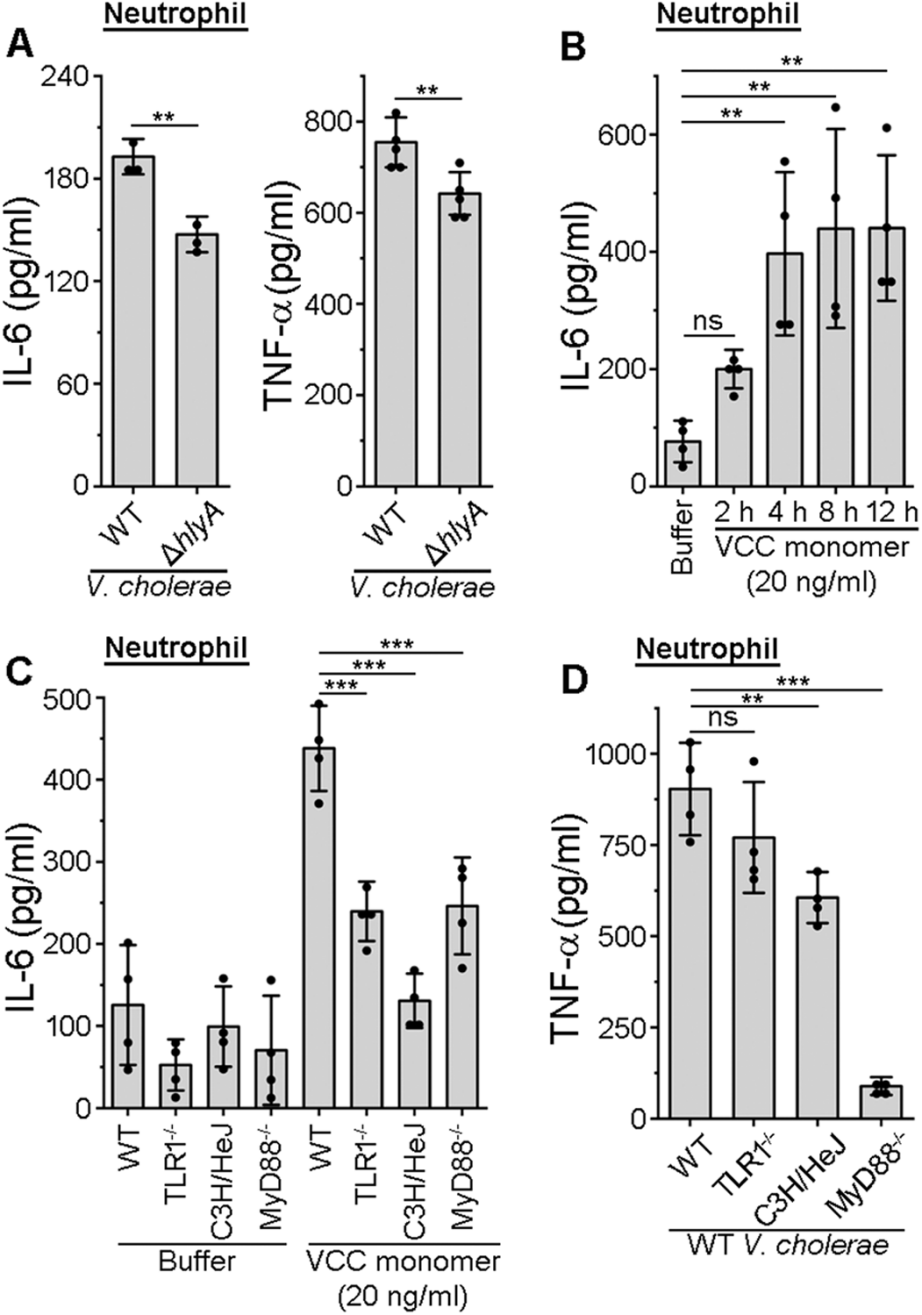
VCC induces activation of neutrophils through TLR1/4/MyD88. (A) VCC induces pro-inflammatory responses during *V. cholerae* infection of neutrophils. Neutrophils isolated from bone marrow were infected with either wild type (WT) or VCC-deleted (Δ*hlyA*) variants of *V. cholerae* at the multiplicity of infection (MOI) of 10 for 4 hours. Supernatants were analysed for the production of IL-6 (left) and TNF-α (right) using ELISA. Data shown here are the averages ± SDs from three to five independent experiments. **, p < 0.01; Student’s unpaired t-test. (B) Increase in the IL-6 production by the neutrophils over time upon VCC treatment. Neutrophils isolated from the bone marrow were treated with 20 ng/ml VCC monomer for 2, 4, 8 and 12 hours. Following treatments, the supernatants were analysed for the IL-6 production. Buffer-treated cells served as the negative control. Data shown here are the averages ± SDs from the four independent experiments. ns, non-significant; **, p < 0.01; one-way ANOVA with Dunnett’s multiple comparison test. (C) Significant decrease in the VCC-mediated IL-6 production in the neutrophils from the TLR1^-/-^, C3H/HeJ, and MyD88^-/-^ mice, as compared to those in neutrophils from the wild type (WT) mice. Neutrophils from the wild type (WT), TLR1^-/-^, C3H/HeJ, and MyD88^-/-^ mice were treated with 20 ng/ml VCC monomer. After 12 hours of treatment, the supernatants were analysed for the IL-6 production using ELISA. Buffer-treated cells served as the negative controls. Data shown here are the averages ± SDs from four independent experiments. ***, p < 0.001; one-way ANOVA with Tukey’s multiple comparison test. (D) VCC-induced pro-inflammatory responses in neutrophils during the *V. cholerae* infection are TLR1, TLR4 and MyD88-dependent. Neutrophils isolated from the bone marrow of the wild type (WT), TLR1^-/-^, C3H/HeJ, and MyD88^-/-^ mice were infected with wild type (WT) *V. cholerae* at an MOI of 10 for 4 hours. Supernatants were analysed for the production of TNF-α using ELISA. Data shown here are the averages ± SDs from four independent experiments. ns, non-significant; **, p < 0.01; ***, p < 0.001; one-way ANOVA with Dunnett’s multiple comparison test.

We observed that VCC could induce the activation of the DCs and macrophages. In addition to DCs and macrophages, neutrophils are another critical responding cells during the *V. cholerae* infection [24,57]. Neutrophils are generally recruited at the site of infection in response to the chemokines, secreted by the macrophages and DCs upon contact with the microbial pathogens [58]. VCC is known to exert cytocidal effects on the neutrophils [59]. To understand whether VCC plays an important role in the neutrophil activation, when infected with *V. cholerae*, we used wild type *V. cholerae* and Δ*hlyA* strain. We isolated neutrophils from the mice bone marrow, and infected with wild type *V. cholerae* and the Δ*hlyA* mutant, and checked for the pro-inflammatory cytokine responses. We observed a diminished IL-6 and TNF-α production by the neutrophils infected with Δ*hlyA* as compared to those infected with the wild type *V. cholerae* (Fig 9A). This result suggested that VCC plays an important role in contributing to the pro-inflammatory responses from the neutrophils, following *V. cholerae* infection. Further, we observed that the treatment of the neutrophils with VCC monomer led to an increase in the cytokine production with an increase in the treatment time from 2 hours up to 12 hours (Fig 9B). Further, we observed that the cytokine responses upon VCC treatment were attenuated, when we used neutrophils isolated from the TLR1^-/-^, MyD88^-/-^, and C3H/HeJ (TLR4-defective) mice, as compared to those from the wild type mice (Fig 9C). These observations confirm that TLR1/4/MyD88 also play an important role in the VCC-mediated activation of the neutrophils. Furthermore, as observed upon treatment with purified VCC, *V. cholerae*-induced cytokine production was also attenuated upon infection in the neutrophils, isolated from the TLR1^-/-^, MyD88^-/-^ and C3H/HeJ (TLR4-defective), mice as compared to those from the wild type mice (Fig 9D). These results emphasize the role TLR1/4 and MyD88 signalling pathway in the activation of neutrophils during the *V. cholerae* infection.

## Discussion

The innate immune system performs the complicated task of differentiating the ‘self’ from the ‘non-self’, and mounting an adequate immune response [60]. Immune cells such as neutrophils, dendritic cells (DCs), macrophages, and monocytes play essential and specific roles in maintaining the homeostasis by eliminating the foreign invaders [61]. These immune cells express several innate receptors or PRRs to carry out the immune surveillance [61, 62]. TLRs are one of the major classes of PRRs that play an important role in executing this function efficiently. TLRs get activated in response to specific PAMPs. Upon recognition of the PAMP, TLRs form either heterodimers or homodimers that, in turn, contribute to their diversity for the pathogen recognition [39,63]. The cooperative interactions among the TLRs, and with different co-receptors, substantially increase the ligand/PAMP recognition diversity [64–66]. A large number of studies have been done till date that have strengthened our understanding regarding the TLRs and their roles in the efficiency of the immune system. However, it is not very clearly understood how these limited numbers of receptors have evolved to recognize the infinite numbers of patterns of several pathogens and damaged self-molecules. In this context, our present study has provided novel insights regarding the employment of different receptor coordination, with TLR being at the centre, for the purpose of recognising a pathogenic bacterium-derived PAMP, VCC, which is a well-known potent PFT.

It has been shown previously that VCC monomer can induce up-regulation of not only TLR2, but that of TLR4 as well, in the peritoneal macrophages [22]. This observation raises some questions, such as, in addition to TLR2, whether TLR4 could also play a role in the VCC-mediated activation of the innate immune cells, and whether more than one TLR could be involved in the recognition of VCC. TLR4 plays a significant role in the recognition of several cholesterol-dependent PFTs, such as Anthrolysin O, Listeriolysin O, and Pneumolysin [67–70]. Therefore, we wanted to know whether VCC could activate DCs, and whether it could engage TLR2 or any other TLRs, such as TLR4 for the activation of DCs. Conventionally, TLR2 heterodimerises with TLR1 and TLR6. Studies have conclusively shown that the heterodimerization of TLR2 with TLR1 or TLR6 has developed evolutionarily to broaden the ligand recognition diversity, and not to evoke distinct immune responses [71]. A novel TLR heterodimer between TLR6 and TLR4 has been documented in response to the endogenous ligands oxLDL and β-amyloid protein [50]. In the present study, we have identified a novel TLR heterodimer formation between TLR1 and TLR4 upon treatment with VCC (Figs 1, 3 and 4). Our present study has shown that in macrophages, in addition to TLR2/6, VCC monomer also engages TLR1 and TLR4 to induce pro-inflammatory responses (Fig 5). Further, previous study with monocytes and macrophages has shown the involvement of TLR2/6 heterodimer in the VCC oligomer-mediated pro-inflammatory responses [10]. Our present study has revealed that in macrophages, in addition to TLR2/6, similar to VCC monomer, VCC oligomer also involves TLR1 and TLR4 for the induction of pro-inflammatory responses (Fig 5). However, in contrast, in DCs, both VCC monomer and oligomer engage only TLR1/4 heterodimer for the induction of pro-inflammatory responses (Figs 1 and 3). Further, in accordance with our previous and present studies with macrophages, we have observed that VCC oligomer is able to stimulate pro-inflammatory signal in the TLR2-TLR6-co-transfected HEK293 cells to some extent (Fig 4B). However, the responses are more prominent in the cells with TLR1-TLR4 co-expression (Fig 4), upon stimulation with both VCC monomer and oligomer, thus suggesting TLR1/4 heterodimer to be the preferential heterodimer for VCC. Such a model of TLR heterodimerization has not been documented previously in response to any endogenous or pathogenic ligand. TLR signalling depends on the nature of the stimulus, the activated TLRs, and the downstream adaptor molecules. Since both VCC monomer and oligomer can activate the similar and novel TLR heterodimer in DCs, the resultant responses would be expected to be similar as well for both the forms of the toxin. However, the extent of the inflammatory cytokine production was found to be considerably less in the case of the VCC monomers, as compared to that for the VCC oligomer. This could be due to the higher cytotoxic activity exerted by the VCC monomer, in comparison to the oligomeric form, which, once generated cannot further exert the membrane-damaging cytotoxic activity. Whatsoever, this unique understanding that different sets of TLRs can be engaged by the same ligand, and vary depending on the different cell types, intrigued us. HEK293 cell co-expression data led us to anticipate that the variation in the receptor dominance in different cell types could possibly lead to differences in the TLR heterodimerization model, which could recognize VCC in a diverse way among the macrophages and DCs. Towards finding out what could be the reason behind it, we checked for the basal level surface expressions of the said TLRs in DCs and macrophages, and further examined for the surface expression of the said TLRs in both the cell types, upon treatment with VCC monomer and oligomer (S5-S7 Figs). Results from these experiments reveal that in DCs, TLR1 and TLR4 are the most available receptors on the surface to accommodate VCC (S5-S7 Figs). However, in macrophages, we have observed increased surface expression of TLR2 and TLR6, and to some extent, TLR4, suggesting that in macrophages TLR2/6 heterodimer is predominantly available to accommodate VCC. In addition, the presence of the basal level TLR4, and a nominal level of TLR1, in the macrophages could also act to mediate VCC-induced signals (S5-S7 Figs). In addition to the differences in the basal level surface expression of the receptors, VCC treatment also led to a change in the TLR expression patterns. These differences might be the result of the different surface expression of these receptors in both the cell types, as well as the specific activation of the downstream pathways triggered upon the VCC treatment. TLRs can express differently among the different immune cells, and respond to the different stimuli. Surface expression of the TLRs at the basal level is low and varies with different cell types. The expression and locations of the TLRs are regulated in response to the specific molecules derived from the pathogens and the damaged host cells. It is well-appreciated that the pathogenic PAMPs can alter the TLR expression levels directly or through the autocrine stimulation by secreted factors such as cytokines [72–75]. Many TLR agonists are known to upregulate or downregulate the TLR levels, which depend on the specific TLRs involved, and the cell type as well. The differential expression of cytokines in the pathological context also impacts the TLR expression pattern, and the responses by the cells [76, 77]. In sum, differential expression of the TLRs on different cells is presumably involved in fine-tuning the host immune responses. Consistent with such notions, similar mechanisms would be expected to be operational in regarding the roles of the TLR expressions for the VCC recognition, and VCC-induced modulation of the TLR expression. However, in this present case, we still cannot specifically comment on what exactly promotes the heterodimerization of these TLRs. Reports suggest that several TLR heterodimers such as TLR2/1, TLR2/6 and TLR2/10 are pre-formed on the cell surface [55]. However, without the ligand binding, the interaction between the intra-cellular moieties of these receptors is hindered. Thus, it does not allow the induction of the intra-cellular signalling pathways. In some cases, the ligand binding can also induce TLR heterodimerization [78]. Different mechanisms of TLR heterodimerization and activation may exist, considering the vast variety of the structurally different TLR ligands.

Involvements of more than one TLR heterodimer have been reported in a few earlier studies. It has been reported earlier for the *Pseudomonas aeruginosa* virulence factor, exoenzyme S (ExoS) [79]. Involvement of both TLR1/2 and TLR2/6 for the engagement *Vibrio parahaemolyticus* OmpU and *Salmonella* Typhimurium OmpV in macrophages has also been reported in the previous studies [37, 38]. Our present study shows that in DCs, VCC engages TLR1/4, but in macrophages, VCC engages both TLR2/6 and TLR1/4. These observations not only reveal a novel TLR heterodimer formation to engage a bacterial ligand, but also show the engagement of more than one TLR heterodimer for the induction/augmentation of the pro-inflammatory responses by the same ligand. Now the question is what could be the necessity of engagement of another TLR heterodimer in the macrophages, compared to that in the DCs. This is probably to augment the pro-inflammatory responses, as it is well-known in the literature that macrophages are more pro-inflammatory in nature. On the other hand, DCs not only contribute to the pro-inflammatory responses, but their major job is antigen presentation to initiate and shape the adaptive responses. Therefore, the pro-inflammatory nature of the DCs is kept in check.

Several TLRs engage co-receptors for their functioning in response to certain ligands. Reports suggest that co-receptors facilitate the assembly, ligand recognition, and function of the TLRs. MD-2 and CD14 act as the co-receptors for TLR4 for the recognition of its iconic ligand LPS. However, we have observed that MD-2 and CD14 are not involved in the VCC-mediated activation of TLR4; rather, only TLR1 is needed, suggesting TLR1 is probably mainly responsible for the ligand recognition, and TLR4 is responsible for mediating the signalling process (Fig 4). Further, no requirement of MD-2 and CD14 also suggests very clearly that the responses are specific to VCC and are not the result of LPS contamination.

A scavenger receptor, CD36, is also widely known to act as a co-receptor with TLR2/6. Further, in the formation of the novel TLR4/6 heterodimer in response to the endogenous ligands, role of CD36 as a co-receptor has been documented in the inside-out signalling [50]. CD36 interaction with DAMPs/PAMPs triggers an intra-cellular signalling cascade resulting in the activation of Src-family kinases, MAPKs, and then subsequent activation of the transcriptional regulators. Notably, the cytosolic tail of CD36 does not have any known signalling motif. However, it can recruit a signalosome complex involving intracellular and membrane protein counterparts. Reports suggest the role of ligand-induced interactions of CD36, as co-receptor, with the TLRs. Additionally CD36 can also interact with Na^+^/K^+^-ATPase, β1/β2 integrins for the signal transduction purpose [80]. During our present study, we wanted to understand if CD36 plays any role in the formation and functioning of the novel TLR1/4 heterodimer. Our investigation suggests that CD36 engages VCC for the activation of DCs in terms of production of pro-inflammatory cytokines (S4 Fig). Further, in the heterologous receptor co-expression assays in the HEK293 cells, we observed that VCC could induce activation of NF-κB to a significant level, when TLR1 and TLR4 are present together, and similarly, when CD36 alone is individually expressed (Figs 4 and S4). It is important to note that, in our study with the BMDCs/BMDMs, the absence of TLR1, TLR4 and MyD88 resulted in the partial decrease in the cytokine responses (Figs 1-3). This implies that CD36 might contribute to the cytokine response significantly in the absence of the TLR1/4/MyD88 signalling axis. Our results with the HEK293 cells further showed that CD36 over-expression alone could induce significant activation of NF-κB upon VCC treatment (S4G-S4H Fig), which signifies that the CD36 activation in response to VCC is possibly independent of the TLRs. Furthermore, TLR1/4-induced NF-κB activation in HEK293 cells upon VCC stimulation also appears to be independent of CD36. These observations suggest that CD36 alone is sufficient to trigger the pro-inflammatory signal, and it is not necessary that CD36 has to work as a co-receptor along with the TLR1/4 heterodimer assembly. Our investigation suggests that the engagement of VCC with CD36 and TLR1/4 possibly triggers additive effect, rather than showing co-operation between these PRRs upon VCC recruitment. This is because the VCC-induced NF-κB activation response upon CD36/TLR1/4 co-expressions in the HEK293 cells is found to be nearly similar to the sum of that in the CD36-expressing cells plus that in the TLR1/4-co-expressing cells. Thus, these results depict that the NF-κB activation could be the result of the additive effect of the two separate signalling pathway activation upon binding of VCC to CD36 and TLR1/4 heterodimer individually (S4 Fig).

We have further observed that, consistent with the results of our in vitro studies, TLR1, TLR4 as well as TLR2 play important roles in the VCC-mediated pro-inflammatory responses in vivo (Fig 6A). However, only TLR1 and TLR4 play crucial role in the VCC-mediated toxicity in vivo (Fig 6D). Further, involvement of MyD88 in both in vivo and in vitro pro-inflammatory response and toxicity induced by VCC suggests that TLR-mediated signalling plays an important role in the functioning of VCC (Figs 6, 8A and 8B).

It has been shown in a very recent study that, when immune cells are infected with the VCC-deleted *V. cholerae* mutant, the extent of cell-death is significantly reduced, in comparison to that observed with the wild type *V. cholerae*-infected cells [56]. This observation has established VCC to be the primary toxin of *V. cholerae* responsible for the host immune cell death following biofilm formation [56]. Our present study reveals that in BMDCs and BMDMs, *V. cholerae* forms biofilms on the cell surface (Fig 7A-7B), and during such biofilm formation VCC acts as a major cell-killing factor secreted by *V. cholerae* (Fig 7C-7D). Further, our study also demonstrates that TLR-mediated signalling is necessary for the *V. cholerae*-mediated cell death in BMDCs and BMDMs (Fig 8C-8D). Our study further suggests the importance of TLR1/4/MyD88-mediated signalling, but not TLR2-mediated signalling, in the *V. cholerae*-induced cell death in DCs (Fig 8C), similar to that observed in the case of VCC-induced cytotoxicity in DCs (Fig 8A). Interestingly, we have observed that TLR2/MyD88-mediated signalling, but not TLR1-dependent signalling, is involved in the *V. cholerae*-induced cytotoxicity in BMDMs (Fig 8D).

It is important to reiterate here that our results have shown the importance of both TLR2-dependent and TLR1/4-dependent signalling in the VCC-induced pro-inflammatory responses in macrophages (Fig 5). In contrast, VCC-induced pro-inflammatory responses in DCs are found to be dependent on the TLR1/4-dependent signalling, and not through TLR2 (Figs 1-3). Consistent with such observations, VCC and *V. cholerae*-induced death of DCs are also found to be dependent on TLR1/4-mediated, and not TLR2-dependent signalling (Fig 8). While the exact requirement of the TLRs for the VCC-induced macrophage cell death remains unclear, *V. cholerae*-induced death in macrophages is clearly dependent on the TLR2 signalling, and not on the TLR1 signalling (Fig 8). Therefore, it is very intriguing that same TLR combination (TLR1/4) probably exerts different functions in different immune cell types: both pro-inflammatory and death signals in DCs, but majorly pro-inflammatory signal in BMDMs.

The oral infection of mice with both wild type and VCC-null mutant of *V. cholerae* (Δ*hlyA*) indicates that VCC plays a significant role in eliciting pro-inflammatory responses during the *V. cholerae* infection (Fig 6B). Further, analyses of the serum IL-6 level following intra-peritoneal administration of VCC in mice suggest that TLR1, TLR4 and TLR2 play major role in the VCC-mediated *in vivo* pro-inflammatory responses (Fig 6A). However, when tested for the VCC-induced *in vivo* toxicity in mice, TLR1/4, but not TLR2, were found to play a major role in mediating the mortality (Fig 6D). Taken together, these observations suggest that the TLR1/4-mediated death signalling, and probably the systemic death of DCs, have more impact on the mortality in response to VCC. Though TLR2 plays an important role in the VCC-induced macrophage cytotoxicity, systemic death signal for macrophages possibly has less impact on the in vivo mortality upon VCC intoxication; rather, TLR2 possibly plays a major role only in the pro-inflammatory responses, in vivo.

Besides DCs and macrophages, VCC can also activate neutrophils during the *V. cholerae* infection, majorly via the TLR1/4/MyD88 signalling axis (Fig 9). These observations highlight the contributions of the different immune cell types in the VCC-mediated inflammation, and the importance of the TLR1/4 heterodimer as a major receptor dimer, critical to different immune cell type function in the *V. cholerae* infection scenario.

TLR signalling pathways induce multitude of cellular responses through activation or suppression of gene expression, including those implicated for the modulation of inflammatory responses. Further, TLR signalling can lead to programmed cell death responses. VCC is a potent inducer of cytotoxicity, and it has been shown to induce apoptosis in the target nucleated cells. Our present study reveals that TLRs play an important role in the VCC-induced cell death in the immune cells. An earlier study has shown that the TLR2 signalling pathway can induce apoptotic signalling in the cells by activating caspase 8 [81]. It has also been shown that TLR4 plays an important role in the induction of apoptosis in macrophages following *Yersinia* infection [82]. Our present study reveals that TLR1/4-mediated signalling plays an important role in the VCC-mediated toxicity. Therefore, the involvement of TLR1/4-mediated signalling could be an important mediator of VCC-induced cell death induction through apoptosis in the immune cells, such as, DCs.

Altogether, our study concludes that VCC activates immune cells leading to the pro-inflammatory cytokine production. This activation is majorly mediated via an unusual heterodimer formation between TLR1 and TLR4, which further leads to the recruitment of the MyD88 adaptor molecule for the initiation of the intra-cellular signalling cascade. VCC-induced responses are found to be also mediated via CD36. However, the TLR1/4 heterodimer formation is independent of the co-receptor help. Further, in macrophages, more than one TLR receptor combination appears to engage VCC as a PAMP for the induction of the pro-inflammatory processes. Our study signifies that there is much yet to be explored in the field of receptor co-operation/co-ordination among the TLRs and/or related PRRs in the context of host-pathogen interaction and immune response generation. Our study further reflects upon the role of TLR-mediated cell death as an important mechanistic contributor towards the pathophysiological function of VCC.

## Materials and Methods

### Ethics statement for the animal experiments

Animal experiments/protocols were approved by the Institutional Animal Ethics Committee (IAEC) of the Indian Institute of Science Education and Research Mohali in accordance with the guidelines issued by the Committee for the Purpose of Control and Supervision of Experiments on Animals (CPCSEA)(Registration no. 1842/GO/ReReBiBt/S/15/CPCSEA). Animals were obtained under the protocols IISERM/SAFE/PRT/2016/006, 2017/007, 2018/010, 2019/001, 2019/011, 2020/008, 2021/013, 2022/022, 2023/020.

### Cell lines and culture conditions

Human embryonic kidney cell line HEK293 (American Type Culture Collection) was maintained in Dulbecco’s Modified Eagle Medium (ThermoFisher Scientific), supplemented with 10% (volume/volume) fetal bovine serum (FBS) (ThermoFisher Scientific), 100 U/ml of penicillin, 100 μg/ml streptomycin (Lonza), at 37 °C in a humidified CO_2_ incubator.

### Purification of VCC

VCC was recombinantly generated and purified using the method described previously [83]. Briefly, the nucleotide sequence encoding precursor form of VCC (pro-VCC) was cloned into a pET-14b vector (Merck Millipore). This construct was transformed into the *Escherichia coli* Origami B cells (Merck Millipore), and protein expression was induced with 1 mM IPTG (B.R. Biochem) at 30 °C for 3 hours. Subsequently, the bacterial cells were pelleted, and were subjected to sonication (Misonix sonicator QSonica, Newton) in the presence of bacterial protease inhibitor (Sigma Aldrich). The recombinant protein was purified from the soluble fraction of the cell lysate by passing through the Ni-NTA Agarose affinity chromatography resin (QIAGEN), followed by Q Sepharose anion-exchange chromatography (Cytiva). For generating mature VCC, the pro-domain was removed by the proteolytic cleavage using trypsin (1:2000 weight/weight ratio of trypsin:protein) at 25 °C for 5 minutes. This reaction mixture was further subjected to Q Sepharose anion-exchange chromatography to obtain the purified form of mature VCC. Protein concentration was estimated by measuring the absorbance at 280 nm, based on the theoretical extinction coefficient derived from the primary structure of the protein.

### Preparation of the pre-formed oligomeric form of VCC

Pre-formed oligomeric form of VCC was generated by incubating the protein in the presence of Asolectin-cholesterol liposomes, following the method described earlier [10]. For the preparation of liposomes, Asolectin (Sigma-Aldrich) and cholesterol (Sigma-Aldrich), at the weight ratio of 1:1, were dissolved in chloroform (Sigma-Aldrich), and dried by evaporation to form a thin film at the bottom of a round-bottom flask. To this lipid film, Phosphate Buffer Saline (PBS; 137 mM NaCl, 2.7 mM KCl, 10 mM Na_2_HPO_4_, 1.8 mM KH_2_PO_4_, pH 7.4) was added to generate the liposome suspension. Large unilamellar liposomes were generated by repeated extrusion through a polycarbonate filter of 100 nm pore size (Avanti Polar lipids). Mature form of VCC monomer was incubated with the freshly prepared liposomes at a protein:lipid weight ratio of 1:3 for 3 hours at 37 °C. Liposome-bound protein was harvested by ultracentrifugation (on a Hitachi Himac CS120GXII micro ultracentrifuge) at 105000 x g for 30 minutes at 4 °C. The pellet fraction was washed 4-5 times with PBS to remove the unbound, monomeric form of VCC. The washed pellet fraction was re-suspended in PBS. Protein concentration was estimated by employing the Bradford reagent (Sigma-Aldrich) assay using BSA (HiMedia) as the standard. SDS-PAGE/Coomassie staining analysis of the preparation, without boiling in the presence of SDS-PAGE sample buffer, confirmed the formation of VCC oligomer in the Asolectin-cholesterol liposomes.

### Isolation of the mouse bone marrow cells

6-8 weeks old BALB/c mice, wild type C57BL/6 mice (WT), transgenic MyD88^-/-^, TLR1^-/-^, TLR2^-/-^, CD36^-/-^ mice, and C3H/HeJ (TLR4^LPS-d^) mice (The Jackson Laboratory) were housed at the Institute Animal Facility. For the isolation of the mouse bone marrow cells, mice were euthanized, sterilized with 70% ethanol, and femur and tibia were removed surgically. The femur and tibia were cleaned thoroughly to get rid of all the muscles, the bones were washed with ice-cold PBS, and sterilized with 70% ethanol for 2-3 minutes. The bones were then re-washed with ice-cold PBS and transferred to RPMI 1640 media (Lonza). The epiphyses were cut using sterile scissors, and ice-cold RPMI media was flushed into the bones using a syringe. Bone marrow cells were collected in the RPMI media.

### Differentiation of the bone marrow-derived dendritic cells (BMDCs)

The cells collected from the mouse bone marrow, as described above, were centrifuged to discard the supernatant, and the harvested cells were treated with ACK lysis buffer (150 mM NH_4_Cl, 10 mM KHCO_3,_ and EDTA 0.1 mM, pH 7.2) for 5 minutes to lyse the erythrocytes. The ACK lysis buffer was then neutralized with FBS, centrifuged again, and the supernatant was discarded. The cells were re-suspended in RPMI 1640 media containing 10% (volume/volume) FBS, 100 U/ml penicillin, 100 μg/ml streptomycin, 1 mM sodium pyruvate (ThermoFisher Scientific), 0.1 mM non-essential amino acids (NEAA) (ThermoFisher Scientific), 30 μM β-mercaptoethanol (Sigma-Aldrich), and 10 ng/ml GM-CSF (Peprotech). These cells were seeded in a tissue culture plates at the density of 1x10^6^ cells/ml, and incubated at 37 °C in a 5% CO_2_ incubator. After every 2 days, floating cells were removed by removing the media, and the cells were supplemented with fresh media with GM-CSF for differentiation for 6 days. On the 7^th^ day, media without GM-CSF were added, and cells were analysed for the marker of differentiation CD11c (BioLegend). The cells, thus differentiated, showed elevated levels of CD11c marker, as compared to the undifferentiated cells. These cells were used as BMDCs for subsequent experiments.

### Differentiation of bone marrow-derived macrophages (BMDMs)

Bone marrow cells were subjected to ACK lysis buffer treatment, as mentioned above. Upon centrifugation, the cell pellet was suspended in RPMI 1640 media, containing 10% (volume/volume) FBS, 100 U/ml penicillin, 100 μg/ml streptomycin, 1 mM sodium pyruvate (ThermoFisher Scientific), 0.1 mM non-essential amino acids (NEAA) (ThermoFisher Scientific), 30 μM β-mercaptoethanol (Sigma-Aldrich), and 30% of M-CSF enriched media from the L929 cells. The cells were seeded at a density of 1x10^6^ cells/ml. Fresh media, containing 30% L929 cell-conditioned media (LCCM) were added to the cells on every alternate day. On day 7, cells were analysed for upregulation of F4/80 (BioLegend), the marker for the macrophages, using flow cytometry. These cells were used as BMDMs for further experiments.

### Isolation of neutrophils from mouse bone marrow

Murine neutrophils were isolated from 6-8 weeks old wild type C57BL/6 (WT), transgenic MyD88^-/-^, TLR1^-/-^, and C3H/HeJ (TLR4^LPS-d^) mice using the method described previously [84]. Briefly, femur and tibia bones were obtained and sterilised using the method described above, and transferred to RPMI 1640 media (Lonza) containing 2 mM filter-sterilised EDTA (Himedia). The epiphyses were cut using sterile scissors, and ice-cold RPMI media containing 2 mM EDTA was flushed into the bones using a syringe. The bones were cut into smaller pieces using the scissors to flush out the cells completely by scraping the inner surface of the bones. The debris were filtered using a 40 μm cell strainer (SPL Life Sciences) and the cells were centrifuged at 1400 rpm for 5 minutes at 4 ^o^C. The pelleted cells were subjected to treatment with 5 ml of 0.2% NaCl (Sigma Aldrich) for 28 seconds to lyse the RBCs, followed by the addition of 5 ml of 1.6% NaCl to restore the osmolarity to 0.9%. The bone marrow cells were then resuspended in ice-cold sterile PBS. In a separate 15-ml falcon, 3 ml of Histopaque 1077 (Sigma Aldrich) was layered slowly above 3 ml of Histopaque 1119 (Sigma-Aldrich). Further, the bone marrow cells were slowly layered on top of Histopaque 1077, and were subjected to centrifugation at 2,000 rpm for 30 minutes at 25 ^o^C with no brakes. The neutrophils at the interface of Histopaque 1119 and Histopaque 1077 were collected in complete RPMI 1640 media, and centrifuged. Upon centrifugation, the cell pellet containing pure and viable neutrophils was suspended in RPMI 1640 media, containing 10% (volume/volume) FBS, and were used for further experiments. The purity was analysed by checking the surface upregulation of Ly-6G (by flow cytometry using the anti-Ly-6G antibody; R&D Systems) and CD11b (by flow cytometry using the anti-CD11b antibody; BD Biosciences), which serve as the markers for the neutrophils.

### Quantification of IL-6, TNF-α and IL-1β

BMDCs/BMDMs were plated at a density of 1x10^6^ cells/ml, and treated with 10 ng/ml VCC monomer or 1 μg/ml pre-formed VCC oligomer, for 24 hours, unless mentioned otherwise. Neutrophils were plated at a density of 1x10^6^ cells/ml, and treated with 20 ng/ml VCC monomer, for 2, 4, 8 and 12 hours, unless mentioned otherwise. Buffer-treated and liposome-treated cells were taken as the negative controls for the experiments with VCC monomer and VCC oligomer, respectively. 30 minutes prior to the treatments, cells were treated with 10 μg/ml Polymyxin B (Sigma-Aldrich), to neutralize the effect of endotoxin contamination, if any. Following the treatments, culture supernatants were analysed for the presence of different cytokines, using the OptEIA ELISA sets (BD Biosciences) as per the manufacturer’s protocol

### LDH-release assay of cytotoxicity

For assessing the cytotoxicity in BMDCs in response to varying concentrations of the VCC variants, BMDCs were plated at a density of 1x10^6^ cells/ml in a 24 well-plate, and treated with VCC monomer (10 ng/ml and 100 ng/ml) or pre-formed VCC oligomer (1 μg/ml and 10 μg/ml) for 24 hours. Buffer and liposome treatments served as the negative controls for the experiments with VCC monomer and VCC oligomer, respectively. The supernatants were collected after 24 hours of treatment. For comparing the cytotoxicity in BMDCs/BMDMs from the wild type and transgenic mice, cells were treated with VCC monomer (10 ng/ml), and supernatants were collected after 12 hours of incubation. LDH release was measured using the CytoTox 96 Non-Radioactivity Cytotoxicity Assay kit (Promega), according to the manufacturer’s protocol. The absorbance was measured at 490 nm on an iMark Microplate Reader (Bio-Rad Laboratories, USA). The %cytotoxicity was calculated with respect to the untreated cells (representing 0% cytotoxicity), and the maximum cytotoxicity induced by the positive control provided in the kit corresponded to the 100% cytotoxicity.

### MTT assay of cell viability

1x10^5^ BMDCs were plated in 100 μl media in a 96-well plate. Cells were treated with VCC monomer (10 ng/ml and 100 ng/ml) or pre-formed VCC oligomer (1 μg/ml and 10 μg/ml) for 24 hours. Buffer and liposome treatments served as the negative controls for the experiments with VCC monomer and VCC oligomer, respectively. Polymyxin B was added 30 minutes prior to each treatment. Cell viability was measured using EZcount MTT cell assay kit (HiMedia), according to the manufacturer’s protocol. Briefly, 10 μl of MTT reagent was added to each well and mixed properly by swirling. After 4 hours, the crystals formed were dissolved using the solubilization buffer provided in the kit, by pipetting. The absorbance was measured at 595 nm. The %cell viability was calculated with respect to the untreated cells (representing 100% cell viability), while media blanks without any cell corresponded to 0% viability.

### Neutralization of TLR

Cells were plated in a 24-well plate at a density of 1x10^6^ cells/ml. Cells were pre-treated for 2 hours with 5 μg/ml TLR4 neutralizing antibody (BioLegend) or the isotype control (BioLegend). After 90 minutes of antibody treatment, cells were pre-treated further with 10 μg/ml Polymyxin B, and then with VCC monomer or pre-formed VCC oligomer. Supernatants were collected after 24 hours, and were analysed for the cytokine production by ELISA.

### siRNA knock-down of TLR1 in BMDCs

BMDCs were plated at a density of 5x10^4^ cells/well, and were transfected using 75 nM ON-TARGETplus SMARTpool small-interfering RNA (siRNA) against mouse TLR1, or with ON-TARGETplus non-targeted siRNA pool (Dharmacon, GE) using FuGENE HD (Promega) at a ratio of 5:1 (FuGENE: siRNA). The transfection mixture was prepared using Opti-MEM (ThermoFisher Scientific). Cells were cultured for 48 hours in the presence of the transfection mixture containing siRNA, and the knock-down was analysed using RT-qPCR. After 48 hours, the cells were pre-treated with 10 μg/ml Polymyxin B for 30 minutes. Further, the cells were stimulated with 1 μg/ml pre-formed VCC oligomer for 12 hours, the supernatant was collected, and IL-6 was quantified by ELISA.

### Gene expression analysis by semi-quantitative PCR

BMDCs, transfected with siRNA and control (as described above), were harvested and washed with PBS twice. Subsequently, RNA from the BMDCs was isolated using the Nucleopore RNA-isolation kit (Genetix, India), according to the manufacturer’s protocol. The cDNA was then synthesized according to the manufacturer’s protocol using the verso-cDNA kit (ThermoFisher Scientific). RT-qPCR was performed using Maxima SYBR Green Master Mix (ThermoFisher Scientific) in a MasterCycler EP Realplex 4 thermal cycler (Eppendorf). The primer sequences were obtained from the Harvard PrimerBank. The relative level of the *Tlr1* gene was calculated as mean fold change over the untreated control. The threshold cycle (C_T_) values of the samples were normalized over the C_T_ values of the respective housekeeping controls. The primers used in the study are detailed below.

*Hprt:*
forward sequence: 5’-TCAGTCAACGGGGGACATAAA-3’
reverse sequence: 5’-GGGGCTGTACTGCTTAACCAG-3’
*β-actin* :
forward sequence: 5’- GGCTGTATTCCCCTCCATCG-3’
reverse sequence: 5’- CCAGTTGGTAACAATGCCATGT-3’

#### *T* *l* *r* *1*

:

forward sequence: 5’- TGAGGGTCCTGATAATGTCCTAC-3’
reverse sequence: 5’- AGAGGTCCAAATGCTTGAGGC-3’

### Whole-cell lysate preparation

Approximately 6×10^6^ cells were used for preparing the whole-cell lysates. After Polymyxin B (10 μg/ml, 30 minutes) treatment, cells were treated with VCC monomer or pre-formed VCC oligomer for different time points, and after the treatment cells were harvested using a cell scraper, and washed twice with ice-cold PBS. Cells were then scraped in PBS and centrifuged in a Multifuge 5417R centrifuge (Eppendorf) at 3500 rpm for 5 minutes. The cell pellet was then re-suspended into 100 μl whole-cell lysis buffer (50 mM Tris-Cl, 150 mM NaCl, 0.1% SDS, and 0.1% Triton-X 100, pH 8.0) supplemented with 1x mammalian protease inhibitor (Sigma-Aldrich). This mixture was sonicated at 10A for 15 seconds with 3 pulses of 5 seconds each. The mixture was then centrifuged at 16000×g for 30 minutes at 4 °C. The supernatant was then collected. Protein concentration was measured in all the samples using the Bradford assay, with BSA (HiMedia) as standard.

### Co-immunoprecipitation studies using whole-cell lysate

Whole-cell lysates were prepared as described above. The lysates were incubated for 3 hours in the presence of 3 μg/ml of specific antibody in a rotating shaker at low speed at 4 °C. To this mixture, 20 μl protein A/G PLUS-agarose beads (Santa Cruz Biotechnology) were added, and incubated overnight. Post-incubation, beads were pelleted and washed thrice using ice-cold whole-cell lysis buffer at 6000×g at 4 °C. Subsequently, 5x SDS-PAGE loading buffer was added to the beads, and heated at 95 °C for 10 minutes. Solubilised proteins were then analysed by SDS-PAGE followed by immunoblotting.

### Immunoblotting

For immunoblotting, samples were subjected to SDS-PAGE, resolved proteins were then transferred onto the PVDF membranes (mdi Membrane Technologies, Bio-Rad Laboratories). The blots were incubated in a blocking solution containing 5% (weight/volume) BSA in TBST (20 mM Tris-HCl buffer, containing 150 mM NaCl, 0.1% Tween 20, pH 7.6) for 1 hour at room temperature or overnight at 4 °C, and probed further with various antibodies. Antibodies for TLR4, TLR1, MyD88, and β-actin were from Santa Cruz Biotechnology. Antibodies for TLR2 and TLR6 were from Cell Signalling Technologies. Anti-CD36 antibody was from R&D Systems. The blots were incubated with the antibodies diluted in the blocking solution at room temperature for 2-3 hours, or at 4 °C overnight. The blots were then washed with TBST, 4-5 times, each for 10-15 minutes, and then incubated with the HRP-conjugated secondary antibody at room temperature for 1 hour. HRP-conjugated anti-mouse and HRP-conjugated anti-rabbit antibodies were from Sigma-Aldrich. HRP-conjugated anti-goat antibody was from Invitrogen. After washing, the blots were developed using Clarity Western ECL substrate (Bio-Rad Laboratories), and the images were acquired on an ImageQuant LAS 4000 (GE Healthcare Life Sciences).

### Flow cytometry-based analyses of the cell-surface receptors

For probing TLR surface expression on the undifferentiated bone marrow-derived cells, bone marrow-derived cells were plated for 7 days without any differentiation factor in the RPMI 1640 media, containing 10% (volume/volume) FBS, 1 mM sodium pyruvate, 0.1 mM non-essential amino acids, 30 μM β-mercaptoethanol. For checking the basal level TLR expressions, BMDCs and BMDMs were left untreated after the differentiation. For the TLR expressions in the BMDMs/BMDCs, upon VCC treatment, approximately 0.75x10^6^ cells were plated at a density of 1x10^6^ cells/ml, and were treated with 10 ng/ml VCC monomer or 1 μg/ml VCC oligomer for different time points. Subsequently, the cells were harvested and washed twice with PBS. The cells were re-suspended in the FACS buffer (PBS containing 1% FBS and 0.1% sodium azide) supplemented with Fc Block (BD Biosciences)(at 1:50 ratio of Fc Block:FACS buffer, volume/volume), and were incubated for 15-20 minutes on ice. After the incubation, cells were stained with antibodies against TLR1, TLR2, TLR4, and TLR6. Briefly, for TLR1 and TLR4 staining, cells were incubated with anti-TLR1 and anti-TLR4 (Santa Cruz Biotechnology) antibodies at 1:200 dilution (volume/volume) in FACS buffer for 40-45 minutes on ice. Further, the cells were washed 2-3 times using the FACS buffer, and stained with Alexa Flour 488-conjugated secondary antibody (1:400, volume/volume; ThermoFisher Scientific) for 30 minutes on ice. For TLR2 and TLR6 staining, cells were stained with FITC-conjugated anti-TLR2 (1:300, volume/volume) (eBiosciences) and APC-conjugated anti-TLR6 (1:300, volume/volume; R&D Systems) antibodies for 40-45 minutes on ice. Cells were washed 2-3 times, re-suspended in PBS. Surface expression of the TLRs was analysed by flow cytometry on a BD Accuri C6 Plus flow cytometer (BD Biosciences).

### Recombinant expression of cell surface receptors in HEK293 cells for the Luciferase reporter assay of NF-κB activation

Nucleotide sequences encoding TLR1, TLR2, TLR4, TLR6, CD36, MD-2, and CD14 from *Mus musculus* were retrieved from NCBI. RNA isolated from the untreated RAW264.7 macrophage cells was used to generate the cDNA using Superscript III reverse transcriptase kit (Invitrogen). Nucleotide sequences for TLR2, TLR4, TLR6, CD36, MD-2, and CD14 were amplified from the cDNA preparation and cloned into the pcDNA3.1(+) expression vector. pcDNA3.1-TLR1 plasmid was from Addgene (Plasmid #13080).

Surface expression of TLR1, TLR2, TLR4, TLR6, and CD36 on the HEK293 cells after transfection with the recombinant construct was monitored by flow cytometry (S2 Fig). Briefly, HEK293 cells were transfected with 0.5 or 1 μg of empty pcDNA3.1(+) vector, or pcDNA3.1(+)-TLR1, or pcDNA3.1(+)-TLR2, or pcDNA3.1(+)-TLR4, or pcDNA3.1(+)-TLR6, or pcDNA3.1(+)-CD36 plasmids using polyethyleneimine (PEI) [kind gift from Dr. Samarjit Bhattacharyya’s laboratory (IISER Mohali)]. After 24 hours of transfection, HEK293 cells were analysed for the surface expression of TLRs and CD36. For the experiments where TLR1 surface expression was monitored upon VCC or LPS treatment, cells were co-transfected with pcDNA3.1(+)-TLR1, pcDNA3.1(+)-TLR4, pcDNA3.1(+)-CD14 and pcDNA3.1(+)-MD-2 together. After 24 hours of transfection, cells were either kept untreated or treated with 100 ng/ml VCC or 200 ng/ml LPS. Following 12 hours, cells were analysed for the surface expression of TLR1. For checking the cell surface expression of the receptors, cells were harvested by trypsinization, and subjected to staining for the specific receptor. The cells were re-suspended in FACS buffer containing human Fc Block (BD Biosciences) at 1:50 (Fc Block:FACS buffer, volume/volume) dilution, and were incubated for 15-20 minutes on ice. For probing TLR1 surface expression, cells were incubated with anti-TLR1 antibody (1:200, volume/volume) (Santa Cruz Biotechnology) for 40-45 minutes, and then with Alexa Flour 488-conjugated secondary antibody (1:400, volume/volume) (Thermo Fisher Scientific) for 30 minutes on ice. For the analyses of TLR2, TLR4, and TLR6 surface expressions, cells were stained with FITC-conjugated anti-TLR2 antibody (1:300, volume/volume) (BioLegend), PE-conjugated anti-TLR4 antibody (1:200, volume/volume) (BioLegend) and APC-conjugated anti-TLR6 antibody (1:50, volume/volume) (R&D Systems), respectively, for 40-45 minutes. For CD36 surface expression, cells were stained with anti-CD36 antibody (1:300, volume/volume) (R&D Systems) for 40 minutes, and then with Alexa Fluor 647-conjugated secondary antibody (1:500, volume/volume) (Abcam) for 30 minutes on ice. Subsequently, surface expression profile of the receptors was analysed either on a BD FACSCalibur Flow Cytometer or BD Accuri C6 Plus Flow Cytometer (BD Biosciences).

For the Luciferase-reporter assay-based detection of NF-κB activation, HEK293 cells were plated at a density of 2.5×10^4^ cells per well in 100 μl DMEM in 96-well flat-bottom plate. The cells were co-transfected with 0.1 μg each of NF-κB reporter plasmid pGL4.1 (Promega, USA) and *Renilla* luciferase plasmid pRL [kind gift from Dr. Rajesh Ramachandran (IISER Mohali)], along with 0.1 μg of empty pcDNA3.1(+) vector, or pcDNA3.1(+) vectors harbouring the designated cell surface receptors (TLR1, TLR2, TLR4, TLR6, CD36, MD-2, CD14) in different combination. For the transfection of pGL4.1 and pRL, Lipofectamine 2000 (Invitrogen) was used as per the manufacturer’s protocol. For the transfection of the pcDNA3.1(+) vectors, PEI was used at a ratio of 1:3 (DNA:PEI) in the serum-free media. After 8 hours of transfection, cells were supplemented with serum-containing DMEM.

For the NF-κB activation assay, after 24 hours of transfection cells were treated with 100 ng/ml VCC monomer, or 1 μg/ml pre-formed VCC oligomer, or 200 ng/ml LPS for 12 hours. For checking the dose-dependent response of VCC monomer, cells were treated with 10, 50, 100 or 200 ng/ml VCC monomer. For experiments where VCC and LPS co-treatment was done, cells were either co-treated with 100 ng/ml VCC monomer and 200 ng/ml LPS together, or pre-treated with VCC for 2 hours, following which LPS was added to the cells. Polymyxin B (10 μg/ml) was added 30 minutes prior to the VCC treatments. For the estimation of NF-κB activation, Dual-Luciferase Assay kit (Promega) was used, as per the manufacturer’s protocol. The luminescence was measured using a plate reader (BMG Biotech, Germany). The NF-κB activation was estimated by measuring the firefly luciferase luminescence, and the transfection efficiency was monitored by measuring the *Renilla* luciferase luminescence. Relative Luminescence Unit (R.L.U.) was calculated by using the firefly and *Renilla* luciferase luminescence. The luminescence corresponding to the NF-κB activation was normalized with respect to that of the *Renilla* luciferase, corresponding to the transfection efficiency.

### Measurement of serum cytokine level and survival assay upon VCC administration in mice

For the measurement of cytokine level in the mice serum in response to VCC, wild type (C57BL/6) mice were injected intra-peritoneally with 2, 5, and 10 μg of VCC monomer (in 100 μl PBS) to standardize the optimal dose of toxin administration (S8 Fig). After determination of the optimal dose, C57BL/6 wild type, and TLR1^-/-^, TLR2^-/-^, C3H/HeJ, and MyD88^-/-^ transgenic mice were administered with 5 μg of VCC (in 100 μl PBS) or an equal volume of PBS as control. After 4 hours of administration, blood was collected using retro-orbital bleeding. The blood was centrifuged at 2000 × g for 10 minutes at room temperature to separate the serum, and the serum IL-6 level was estimated by ELISA.

For the survival assay, wild type (C57BL/6) and TLR1^-/-^, TLR2^-/-^, C3H/HeJ, and MyD88^-/-^ transgenic mice were injected intra-peritoneally with 5 μg of VCC (in 100 μl PBS) or equal volume of PBS as control. Mice were observed for survival up to 48 hours after VCC intoxication. A Kaplan-Meier plot for cumulative mortality was presented to compare the survival rate of mice from each group.

### Oral infection in mice with *V. cholerae*

Wild type and VCC-deleted (Δ*hlyA*) variants of *V. cholerae* O1 biovar El Tor strain C6706 (kindly provided by Dr. Knut Drescher, Biozentrum, University of Basel, 4056 Basel, Switzerland) were used in this experiment. Towards this, 5-6 weeks-old wild type (C57BL/6) and TLR1^-/-^, C3H/HeJ, and MyD88^-/-^ transgenic mice were used. The mice were kept in fasting for 8 hours before orally administering 50 mg streptomycin and 25 mg kanamycin (in 500 μl sterile water) per mouse. After the antibiotic administration for 2 consecutive days, mice were again kept in fasting for 8 hours and fed with 8.5% (weight/volume) sodium bicarbonate (Sigma-Aldrich) (in 100 μl sterile water) immediately followed by a challenge with wild type or Δ*hlyA* variants of *V. cholerae* (5x10^9^ bacteria per mouse in 500 μl PBS). These mice were further assessed for the bacterial colonization and serum cytokine levels. The serum cytokine levels were measured 4 hours post-infection using the protocol described above.

To assess the bacterial colonization in the mouse gut, bacterial shedding in the stool was measured. For this, bacterial stool was collected and dissolved in sterile PBS. Serial dilutions were plated on TCBS agar (Himedia) plates, and bacterial counts were estimated by calculating the colony-forming unit (CFU) per gram of stool. The colonization of both the variants of bacteria was checked from day 1 to day 10, post-infection.

### Co-culture of immune cells with *V. cholerae*

*V. cholerae* O1 biovar El Tor strain N16961 was obtained from the Microbial Type Culture Collection and Gene Bank of the Institute of Microbial Technology, Chandigarh, India (MTCC code 3905). Wild type and VCC-deleted (Δ*hlyA*) variants of *V. cholerae* O1 biovar El Tor strain C6706 were kindly provided by Dr. Knut Drescher, Biozentrum, University of Basel, 4056 Basel, Switzerland. Culture condition was followed as mentioned in an earlier study [56]. Briefly, bacteria were grown in LB medium supplemented with streptomycin (50 μg/ml) till the exponential growth phase. Cells were then diluted 500-fold and grown in DMEM, supplemented with serum and antibiotic till the stationary phase. For co-culturing, 0.3×10^6^ BMDCs or BMDMs were plated on coverslips for differentiation. At day 7, cells were replenished with fresh media without the differentiation factors. Cells were co-cultured with *V. cholerae* strain N16961 at a multiplicity of infection (MOI) 50 for BMDCs, and 40 for BMDMs for 7 hours at 37 ^o^C. For experiments where wild type and VCC-deleted (Δ*hlyA*) variants of *V. cholerae* strain C6706 were used, MOI 40 and 30 was used for BMDCs and BMDMs, respectively, for 7 hours at 37 ^o^C. For experiments with neutrophils, wild type and VCC-deleted (Δ*hlyA*) variants of *V. cholerae* strain C6706 were used at an MOI of 10 for 4 hours at 37 ^o^C. Following co-culturing of the neutrophils with *V. cholerae*, supernatant was collected and analysed for the cytokine production by ELISA.

### Confocal microscopy to monitor cell death induced by *V. cholerae*

BMDCs/BMDMs were stained with 1 μM CellTrace Far Red dye (Invitrogen, ThermoFisher Scientific) for 20-30 minutes in the serum-free media. Following staining, BMDCs/BMDMs were co-cultured with *V. cholerae* according to the conditions described above. Following infection, cells were treated with Gentamicin (100 μg/ml) (Himedia) for 1 hour, and washed 2-3 times with media. To stain the dead/dying cell population, cells were incubated with 8 μM Propidium Iodide (PI) (BD Pharmingen) for 15 minutes. Subsequently, the cells were washed 2-3 times with PBS, BMDCs were fixed with 2% paraformaldehyde (PFA) (Sigma-Aldrich), and BMDMs were fixed with 4% PFA, followed by 2-3 wash with PBS. The coverslips were then mounted using fluoromount (Sigma-Aldrich) for confocal microscopy. Images were acquired on a Leica confocal laser scanning microscope.

A total of ~15-20 images were taken for each treatment from 3-4 independent sets. Image analysis was done using the ImageJ software (National Institutes of Health, Bethesda, MD, USA). For the preparation of the representative images, images were set to similar brightness and contrast throughout all the samples in a single experiment.

For quantification, maximum intensity projection (MIP) image was generated. This image was then split into red (PI) and green (CellTrace) channel. For the red channel, regions in the image, where bacterial cells were present, were excluded to avoid the false positives. For this, morphological operations were performed in the ImageJ software that included only the round dead cells’ nuclei and excluded the bacteria stained with PI. Subsequently, thresholding was done using equal values for each channel across all the conditions in a particular experimental set, and manual counting was done for the total cells (stained with CellTrace) as well as the dead cells (stained with PI), and %cell death was calculated accordingly.

### Confocal microscopy to monitor biofilm formation by *V. cholerae* on the immune cells

*V. cholerae* O1 biovar El Tor strain C6706 was used in this assay. This strain was transformed with pNUT542 plasmid [isolated from the VCC-deleted C6706 strain (Δ*hlyA*) obtained from Dr. Knut Drescher, Biozentrum, University of Basel, 4056 Basel, Switzerland], a GFP-carrying plasmid. To visualize the biofilm formation, bacteria were grown in LB medium supplemented with gentamicin (30 μg/ml) till the exponential growth phase. BMDCs and BMDMs were stained with 1 μM CellTrace Far Red dye (Invitrogen, ThermoFisher Scientific) and were co-cultured with *V. cholerae* strain C6706 at a multiplicity of infection (MOI) 64 for BMDCs and BMDMs for 2, 4 and 7 hours at 37 °C. Following infection, the cells were washed 2-3 times with PBS, BMDCs were fixed with 2% paraformaldehyde (PFA) (Sigma-Aldrich), and BMDMs were fixed with 4% PFA, followed by 2-3 wash with PBS. The coverslips were then mounted using fluoromount (Sigma-Aldrich) for confocal microscopy. Images were acquired on a Leica confocal laser scanning microscope.

### Statistical analyses

For the comparison between two groups, statistical analyses were performed using the Student’s unpaired *t*-test to determine the p-values using GraphPad QuickCalcs software available online. For the comparison between 3 or more groups, One-way ANOVA followed by Dunnett’s or Tukey’s multiple comparison tests were performed using GraphPad Prism 10. Differences were considered to be statistically significant at the p-values < 0.05, while the p-values > 0.05 were considered as non-significant (ns). *, **, and *** represent p-values < 0.05, < 0.01, and < 0.001, respectively.

## Supporting information

S1 Fig VCC monomer and pre-formed oligomer induce pro-inflammatory responses in the BMDCs. (A) Dose-dependent increase in cytotoxicity in BMDCs upon treatment with VCC monomer (left panel), and pre-formed VCC oligomer generated in the liposomes (right panel). BMDCs were treated with VCC monomer (10 ng/ml and 100 ng/ml) or VCC oligomer (1 μg/ml and 10 μg/ml) for 24 hours. Following the incubation, cytotoxicity was measured by the LDH-release assay. Buffer-treatment and liposome-treatment served as the negative controls for the VCC monomer and VCC oligomer-mediated cytotoxicity measurements, respectively. Data shown here are the averages ± standard deviations (SDs) of %cytotoxicity from four to five independent treatments. **, p < 0.01; ***, p < 0.001; one-way ANOVA with Dunnett’s multiple comparison test. (B) Dose-dependent decrease in the viability of BMDCs upon treatment with VCC monomer (left panel), and pre-formed VCC oligomer (right panel). BMDCs were treated with VCC monomer (10 ng/ml and 100 ng/ml) or VCC oligomer (1 μg/ml and 10 μg/ml) for 24 hours. Following incubation, cell viability was measured using the MTT assay. Bar graphs represent the averages (of %cell viability) ± SDs from three independent treatments. **, p < 0.01; ***, p < 0.001; one-way ANOVA with Dunnett’s multiple comparison test. (C-E) Significant productions of IL-6 (C), TNF-α (D), and IL-1β (E) were observed in BMDCs upon treatment with VCC monomer and pre-formed VCC oligomer. Cells were treated with VCC monomer (10 ng/ml; left panels) or pre-formed VCC oligomer (1 μg/ml; right panels) for 24 hours. Following 24 hours of treatment, cell culture supernatants were collected, and the cytokine productions were estimated by ELISA. Buffer and liposome-treated cells served as the negative controls for the VCC monomer and VCC oligomer treatments, respectively. Data shown here are the averages ± SDs from three to four independent experiments. *, p < 0.05; **, p < 0.01; ***, p < 0.001; Student’s unpaired *t*-test.
(TIF)

S2 Fig Surface expression analyses of TLR1, TLR2, TLR4, TLR6, and CD36 upon transfection of the recombinant constructs in HEK293 cells. (A-E) HEK293 cells were transfected with pcDNA3.1(+) vector alone, or with pcDNA3.1(+)-TLR1 (A), pcDNA3.1(+)-TLR2 (B), pcDNA3.1(+)-TLR4 (C), and pcDNA3.1(+)-TLR6 (D), and pcDNA3.1(+)-CD36 (E) separately. After 24 hours of transfection, cells were subjected to flow cytometry analyses to probe surface expression of the receptors. Untreated unstained (for TLR6), untransfected stained (for CD36, TLR1, TLR2, and TLR4), and pcDNA3.1(+) empty vector-transfected stained cells were taken as the controls. Data shown here are the representatives of one to three independent experiments. (F) Dose-dependent increase in the NF-κB activation upon VCC treatment in the HEK293 cells transfected with TLR1/4. HEK293 cells were transfected with NF-κB Luciferase reporter plasmid, pRL (renilla) plasmid, along with pcDNA3.1(+)-TLR1, pcDNA3.1(+)-TLR4, and empty pcDNA3.1(+), in different combinations. Following 24 hours of transfection, cells were treated with 10, 50, 100 and 200 ng/ml VCC monomer, and the NF-κB reporter activity was measured after 12 hours of the treatments. Empty pcDNA3.1(+)-transfected, untreated cells served as the controls. Data shown here are the averages ± SDs from five independent experiments. ns, non-significant; ***, p < 0.001; one-way ANOVA with Dunnett’s multiple comparison test.
(TIF)

S3 Fig VCC co-treatment attenuates LPS-induced signalling. (A-B) Flow cytometry data showing surface expression of TLR1 in HEK293 cells transfected with pcDNA3.1(+)-TLR1, pcDNA3.1(+)-TLR4, pcDNA3.1(+)-CD14 and pcDNA3.1(+)-MD-2 together, upon treatment with either LPS (A) or VCC (B). The extent of surface expression was quantitated by calculating the %Median Fluorescence Intensity (%MFI) with respect to that from the untreated cells (corresponding to 100%), and the averages ± SDs (from three independent experiments) are shown in the form of bar graphs (right panels in A-B). ns, non-significant; **, p < 0.01; Student’s unpaired *t*-test. (C-D) HEK293 cells were transfected with NF-κB Luciferase reporter plasmid, pRL (renilla) plasmid, along with pcDNA3.1(+)-TLR1, pcDNA3.1(+)-TLR4, pcDNA3.1(+)-CD14, pcDNA3.1(+)-MD-2, and empty pcDNA3.1(+), in different combinations. Following 24 hours of transfection, cells were treated with 100 ng/ml VCC monomer or 200 ng/ml LPS separately (shown in C). In a separate assay, cells were either co-treated with 100 ng/ml VCC monomer and 200 ng/ml LPS together, or pre-treated with 100 ng/ml VCC for two hours, followed by treatment with 200 ng/ml LPS (shown in D). NF-κB reporter activity was measured after 12 hours of treatments. Empty pcDNA3.1(+)-transfected, VCC or LPS-treated cells served as the controls. Data shown here are the averages ± SDs from four (C) to five (D) independent experiments. ns, non-significant; ***, p < 0.001; one-way ANOVA with Tukey’s multiple comparison test.
(TIF)

S4 Fig CD36 is involved in the VCC-induced pro-inflammatory responses in BMDCs. (A-D) Immunoblot analyses showing CD36 co-immunoprecipitation with VCC in BMDCs, treated with 10 ng/ml VCC monomer (A, C) or 1 μg/ml pre-formed VCC oligomer (B, D) for 30 minutes. Whole-cell lysates from the VCC-treated cells were immunoprecipitated (IP) with either anti-VCC antibody (A, B) or anti-CD36 antibody (C, D). The immunoprecipitated fractions were probed by immunoblotting (IB) with anti-VCC and anti-CD36 antibodies. β-actin was probed as the protein loading control in the co-immunoprecipitation assays with anti-VCC antibody. Buffer and liposome-treated cells served as the negative controls for the experiments with VCC monomer and oligomer, respectively. Immunoblots shown here are representatives of three independent experiments. (E-F) Significant decrease in the VCC-mediated IL-6 production in BMDCs from CD36^-/-^ mice, as compared to that in the BMDCs from wild type (WT) mice. BMDCs from CD36^-/-^ mice and wild type (WT) mice were treated with 10 ng/ml VCC monomer (E) and 1 μg/ml pre-formed VCC oligomer (F) for 30 minutes. Following incubation, supernatants were probed for the IL-6 production by ELISA. Buffer and liposome-treated cells served as the negative controls for the experiments with VCC monomer and oligomer, respectively. Data shown here are the averages ± SDs from three independent experiments. **, p < 0.01; ***, p < 0.001; one-way ANOVA with Tukey’s multiple comparison test. (G-H) TLR1-TLR4 hetero-dimer-induced NF-κB activation in response to VCC was possibly independent of CD36. HEK293 cells were transfected with NF-κB Luciferase reporter plasmid, pRL (renilla) plasmid, along with pcDNA3.1(+)-CD36, pcDNA3.1(+)-TLR1, pcDNA3.1(+)-TLR4, and empty pcDNA3.1(+), in different combinations. Following 24 hours of transfection, cells were treated with 100 ng/ml VCC monomer (G) or 1 μg/ml pre-formed VCC oligomer (H), and the NF-κB reporter activity was measured after 12 hours of treatments, as described in Fig 4. Empty pcDNA3.1(+)-transfected, VCC-treated cells served as the controls. Data shown here are the averages ± SDs from three independent experiments. *, p < 0.05; **, p < 0.01; ***, p < 0.001; one-way ANOVA with Dunnett’s multiple comparison test.
(TIF)

S5 Fig Differential surface expression profiles of TLR1, TLR2, TLR4, and TLR6 in BMDCs and BMDMs, in comparison to the undifferentiated bone marrow-derived cells. Differentiation of the bone marrow-derived cells into BMDMs led to a prominent increase in the surface expression of TLR2, TLR4, as well as TLR6, and no noticeable increase in the TLR1 surface expression. In contrast, differentiation into the BMDCs induced a significant increase in the surface expression of TLR1 and TLR4, and some moderate increase in the TLR2 and TLR6. Flow cytometry-based assay was employed to probe the surface expressions of different TLRs, and the representative histogram plots are shown (A-D). Extent of surface expression was quantitated by calculating the %Median Fluorescence Intensity (%MFI) with respect to that from the undifferentiated cells (corresponding to 100%), and the averages ± SDs (from three to six independent experiments) are shown in the form of bar graphs (A-D). ns, non-significant; *, p < 0.05; **, p < 0.01; one-way ANOVA with Dunnet’s multiple comparison test.
(TIF)

S6 Fig VCC oligomer treatment up-regulates TLR2 and TLR6 surface expression in BMDMs, and TLR1 and TLR4 surface expression in BMDCs. (A-D) Flow cytometry-based data showing changes, if any, in the surface expression of TLR1 (A), TLR2 (B), TLR4 (C), and TLR6 (D) upon treatment of BMDMs or BMDCs with VCC oligomer (1 μg/ml; for the specified time periods). Subsequently, TLR expressions were monitored by flow cytometry. Liposome-treated cells served as the negative controls. Offset histograms (shown in A-D) are the representatives of three to four independent experiments. Extent of TLR surface expressions was quantitated by calculating the %Median Fluorescence Intensity (%MFI) with respect to that from the liposome-treated cells (corresponding to 100%), and the averages ± SDs (from three to four independent experiments) are shown in the form of bar graphs (A-D). *, p < 0.05; one-way ANOVA with Dunnet’s multiple comparison test.
(TIF)

S7 Fig VCC monomer induces increased TLR2 and TLR6 surface expression in BMDMs, and increased TLR1 and TLR4 surface expression in BMDCs. (A-D) Flow cytometry-based data showing changes, if any, in the surface expression of TLR1 (A), TLR2 (B), TLR4 (C), and TLR6 (D) upon treatment of BMDMs or BMDCs with VCC monomer (10 ng/ml; for the specified time periods). Subsequently, TLR expressions were monitored by flow cytometry. Buffer-treated cells served as the negative controls. Offset histograms (shown in A-D) are the representatives of three to four independent experiments. Extent of TLR surface expressions was quantitated by calculating the %Median Fluorescence Intensity (%MFI) with respect to that from the buffer-treated cells (corresponding to 100%), and the averages ± SDs (from three to four independent experiments) are shown in the form of bar graphs (A-D). *, p < 0.05; one-way ANOVA with Dunnet’s multiple comparison test.
(TIF)

S8 Fig Measurement of serum IL-6 level upon intra-peritoneal administration of different doses of VCC in mice. C57BL/6 mice were injected intra-peritoneally with 2, 5, and 10 μg VCC (in 100 μl PBS), and blood was collected after 4 hours. Serum was obtained, and IL-6 level in the serum was estimated by ELISA. Mice injected with equal volume of PBS served as the negative controls. Data shown here are the averages ± SDs from three independent experiments. ***, p < 0.001; one-way ANOVA with Dunnett’s multiple comparison test.
(TIF)

S1 Data Source data for the graphs in Figs 1-9 and S1-S8. (XLSX)
