# Supplementary figures and images for: Vibrio cholerae cytolysin induces pro-inflammatory and death signals through novel TLR assembly
Source: PLoS Pathog. 2025 Apr 4;21(4):e1013033. doi: 10.1371/journal.ppat.1013033 (PMC12002540; doi:10.1371/journal.ppat.1013033)

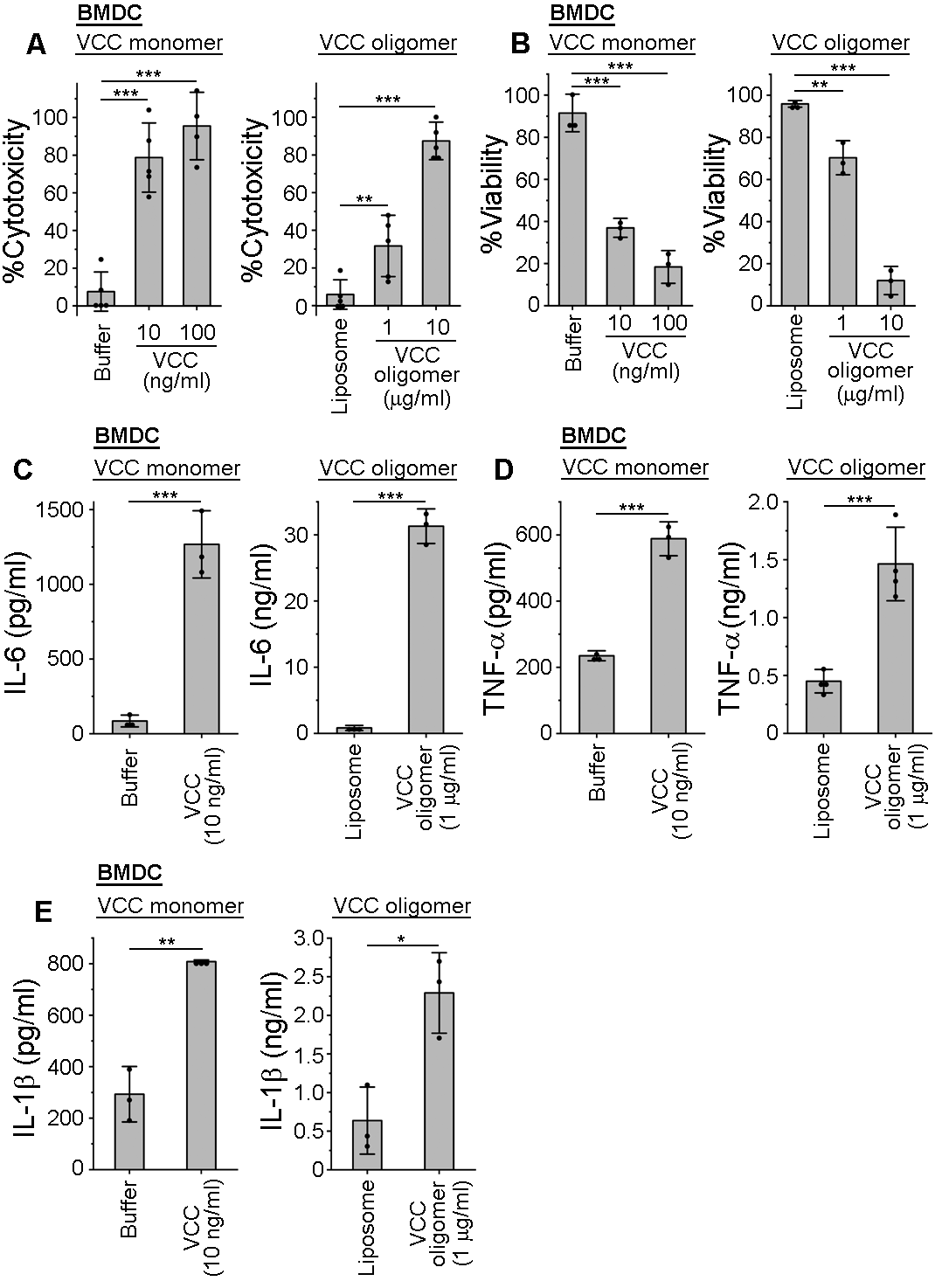

Supplement: S1 Fig — (A) Dose-dependent increase in cytotoxicity in BMDCs upon treatment with VCC monomer (left panel), and pre-formed VCC oligomer generated in the liposomes (right panel). BMDCs were treated with VCC monomer (10 ng/ml and 100 ng/ml) or VCC oligomer (1 μg/ml and 10 μg/ml) for 24 hours. Following the incubation, cytotoxicity was measured by the LDH-release assay. Buffer-treatment and liposome-treatment served as the negative controls for the VCC monomer and VCC oligomer-mediated cytotoxicity measurements, respectively. Data shown here are the averages ± standard deviations (SDs) of %cytotoxicity from four to five independent treatments. **, p < 0.01; ***, p < 0.001; one-way ANOVA with Dunnett’s multiple comparison test. (B) Dose-dependent decrease in the viability of BMDCs upon treatment with VCC monomer (left panel), and pre-formed VCC oligomer (right panel). BMDCs were treated with VCC monomer (10 ng/ml and 100 ng/ml) or VCC oligomer (1 μg/ml and 10 μg/ml) for 24 hours. Following incubation, cell viability was measured using the MTT assay. Bar graphs represent the averages (of %cell viability) ± SDs from three independent treatments. **, p < 0.01; ***, p < 0.001; one-way ANOVA with Dunnett’s multiple comparison test. (C-E) Significant productions of IL-6 (C), TNF-α (D), and IL-1β (E) were observed in BMDCs upon treatment with VCC monomer and pre-formed VCC oligomer. Cells were treated with VCC monomer (10 ng/ml; left panels) or pre-formed VCC oligomer (1 μg/ml; right panels) for 24 hours. Following 24 hours of treatment, cell culture supernatants were collected, and the cytokine productions were estimated by ELISA. Buffer and liposome-treated cells served as the negative controls for the VCC monomer and VCC oligomer treatments, respectively. Data shown here are the averages ± SDs from three to four independent experiments. *, p < 0.05; **, p < 0.01; ***, p < 0.001; Student’s unpaired t-test. (TIF) [file ppat.1013033.s001.tif]

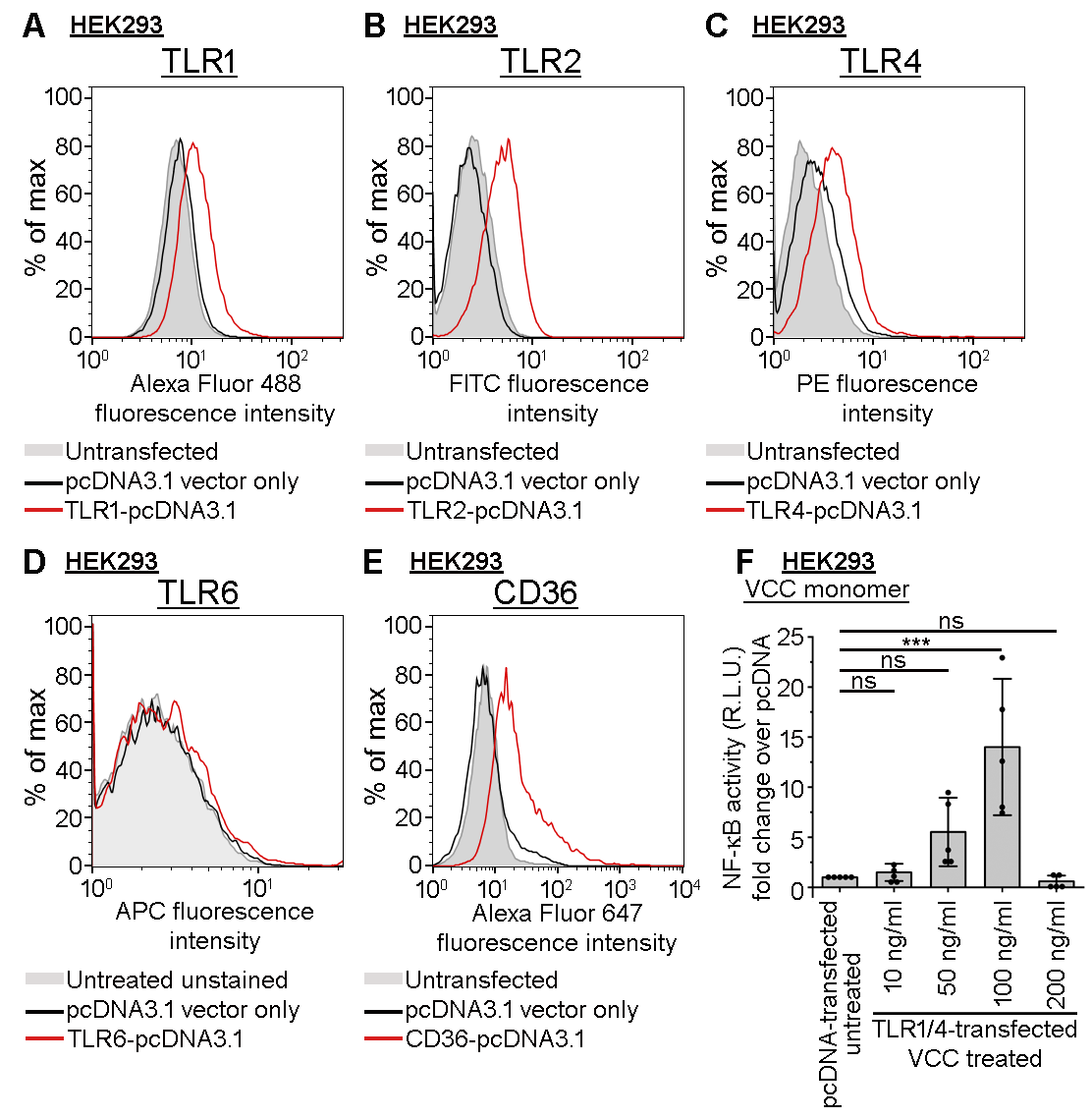

Supplement: S2 Fig — (A-E) HEK293 cells were transfected with pcDNA3.1(+) vector alone, or with pcDNA3.1(+)-TLR1 (A), pcDNA3.1(+)-TLR2 (B), pcDNA3.1(+)-TLR4 (C), and pcDNA3.1(+)-TLR6 (D), and pcDNA3.1(+)-CD36 (E) separately. After 24 hours of transfection, cells were subjected to flow cytometry analyses to probe surface expression of the receptors. Untreated unstained (for TLR6), untransfected stained (for CD36, TLR1, TLR2, and TLR4), and pcDNA3.1(+) empty vector-transfected stained cells were taken as the controls. Data shown here are the representatives of one to three independent experiments. (F) Dose-dependent increase in the NF-κB activation upon VCC treatment in the HEK293 cells transfected with TLR1/4. HEK293 cells were transfected with NF-κB Luciferase reporter plasmid, pRL (renilla) plasmid, along with pcDNA3.1(+)-TLR1, pcDNA3.1(+)-TLR4, and empty pcDNA3.1(+), in different combinations. Following 24 hours of transfection, cells were treated with 10, 50, 100 and 200 ng/ml VCC monomer, and the NF-κB reporter activity was measured after 12 hours of the treatments. Empty pcDNA3.1(+)-transfected, untreated cells served as the controls. Data shown here are the averages ± SDs from five independent experiments. ns, non-significant; ***, p < 0.001; one-way ANOVA with Dunnett’s multiple comparison test. (TIF) [file ppat.1013033.s002.tif]

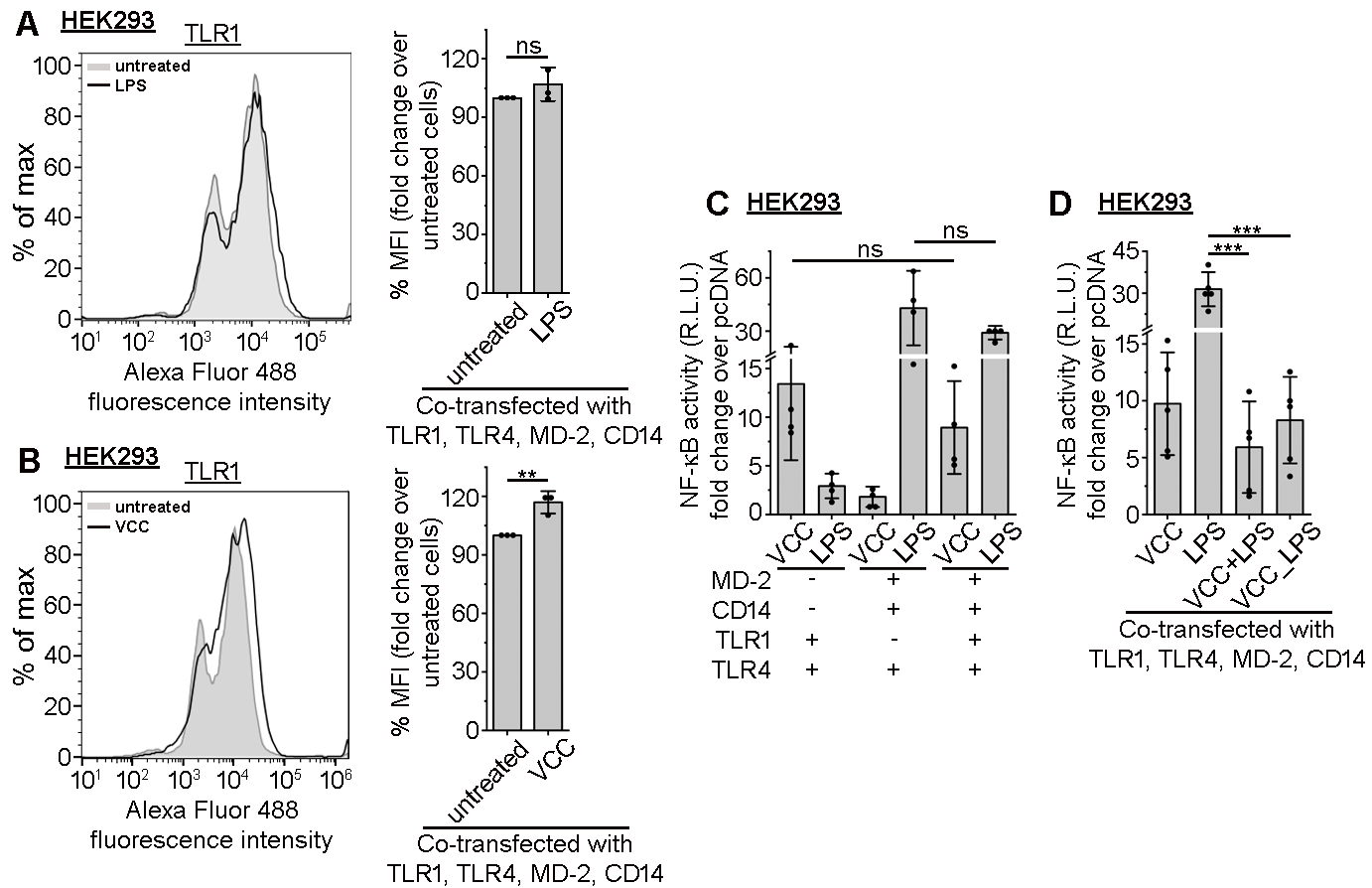

Supplement: S3 Fig — (A-B) Flow cytometry data showing surface expression of TLR1 in HEK293 cells transfected with pcDNA3.1(+)-TLR1, pcDNA3.1(+)-TLR4, pcDNA3.1(+)-CD14 and pcDNA3.1(+)-MD-2 together, upon treatment with either LPS (A) or VCC (B). The extent of surface expression was quantitated by calculating the %Median Fluorescence Intensity (%MFI) with respect to that from the untreated cells (corresponding to 100%), and the averages ± SDs (from three independent experiments) are shown in the form of bar graphs (right panels in A-B). ns, non-significant; **, p < 0.01; Student’s unpaired t-test. (C-D) HEK293 cells were transfected with NF-κB Luciferase reporter plasmid, pRL (renilla) plasmid, along with pcDNA3.1(+)-TLR1, pcDNA3.1(+)-TLR4, pcDNA3.1(+)-CD14, pcDNA3.1(+)-MD-2, and empty pcDNA3.1(+), in different combinations. Following 24 hours of transfection, cells were treated with 100 ng/ml VCC monomer or 200 ng/ml LPS separately (shown in C). In a separate assay, cells were either co-treated with 100 ng/ml VCC monomer and 200 ng/ml LPS together, or pre-treated with 100 ng/ml VCC for two hours, followed by treatment with 200 ng/ml LPS (shown in D). NF-κB reporter activity was measured after 12 hours of treatments. Empty pcDNA3.1(+)-transfected, VCC or LPS-treated cells served as the controls. Data shown here are the averages ± SDs from four (C) to five (D) independent experiments. ns, non-significant; ***, p < 0.001; one-way ANOVA with Tukey’s multiple comparison test. (TIF) [file ppat.1013033.s003.tif]

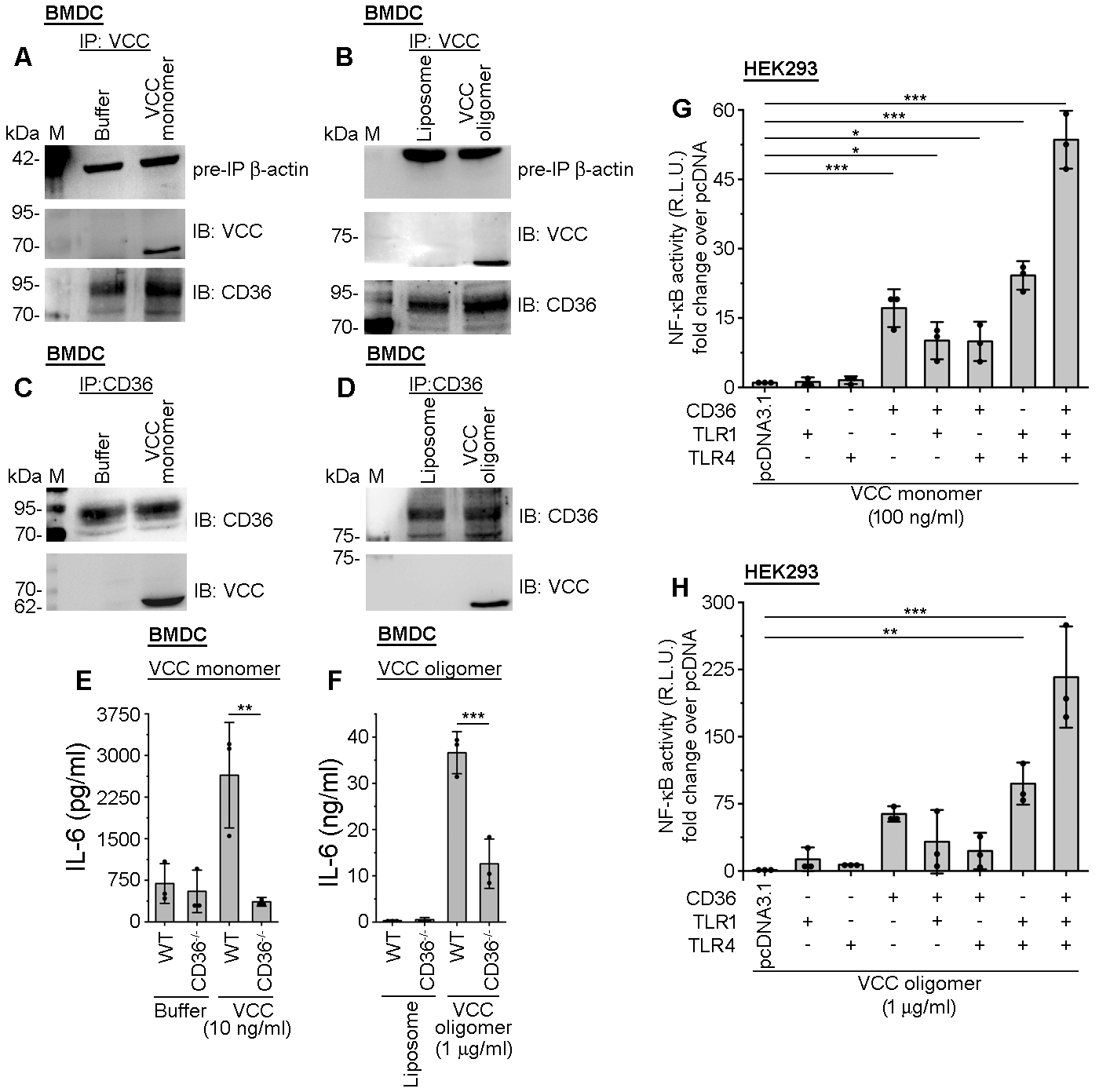

Supplement: S4 Fig — (A-D) Immunoblot analyses showing CD36 co-immunoprecipitation with VCC in BMDCs, treated with 10 ng/ml VCC monomer (A, C) or 1 μg/ml pre-formed VCC oligomer (B, D) for 30 minutes. Whole-cell lysates from the VCC-treated cells were immunoprecipitated (IP) with either anti-VCC antibody (A, B) or anti-CD36 antibody (C, D). The immunoprecipitated fractions were probed by immunoblotting (IB) with anti-VCC and anti-CD36 antibodies. β-actin was probed as the protein loading control in the co-immunoprecipitation assays with anti-VCC antibody. Buffer and liposome-treated cells served as the negative controls for the experiments with VCC monomer and oligomer, respectively. Immunoblots shown here are representatives of three independent experiments. (E-F) Significant decrease in the VCC-mediated IL-6 production in BMDCs from CD36-/- mice, as compared to that in the BMDCs from wild type (WT) mice. BMDCs from CD36-/- mice and wild type (WT) mice were treated with 10 ng/ml VCC monomer (E) and 1 μg/ml pre-formed VCC oligomer (F) for 30 minutes. Following incubation, supernatants were probed for the IL-6 production by ELISA. Buffer and liposome-treated cells served as the negative controls for the experiments with VCC monomer and oligomer, respectively. Data shown here are the averages ± SDs from three independent experiments. **, p < 0.01; ***, p < 0.001; one-way ANOVA with Tukey’s multiple comparison test. (G-H) TLR1-TLR4 hetero-dimer-induced NF-κB activation in response to VCC was possibly independent of CD36. HEK293 cells were transfected with NF-κB Luciferase reporter plasmid, pRL (renilla) plasmid, along with pcDNA3.1(+)-CD36, pcDNA3.1(+)-TLR1, pcDNA3.1(+)-TLR4, and empty pcDNA3.1(+), in different combinations. Following 24 hours of transfection, cells were treated with 100 ng/ml VCC monomer (G) or 1 μg/ml pre-formed VCC oligomer (H), and the NF-κB reporter activity was measured after 12 hours of treatments, as described in Fig 4. Empty pcDNA3.1(+)-transfected, VCC-treated ce [file ppat.1013033.s004.tif]

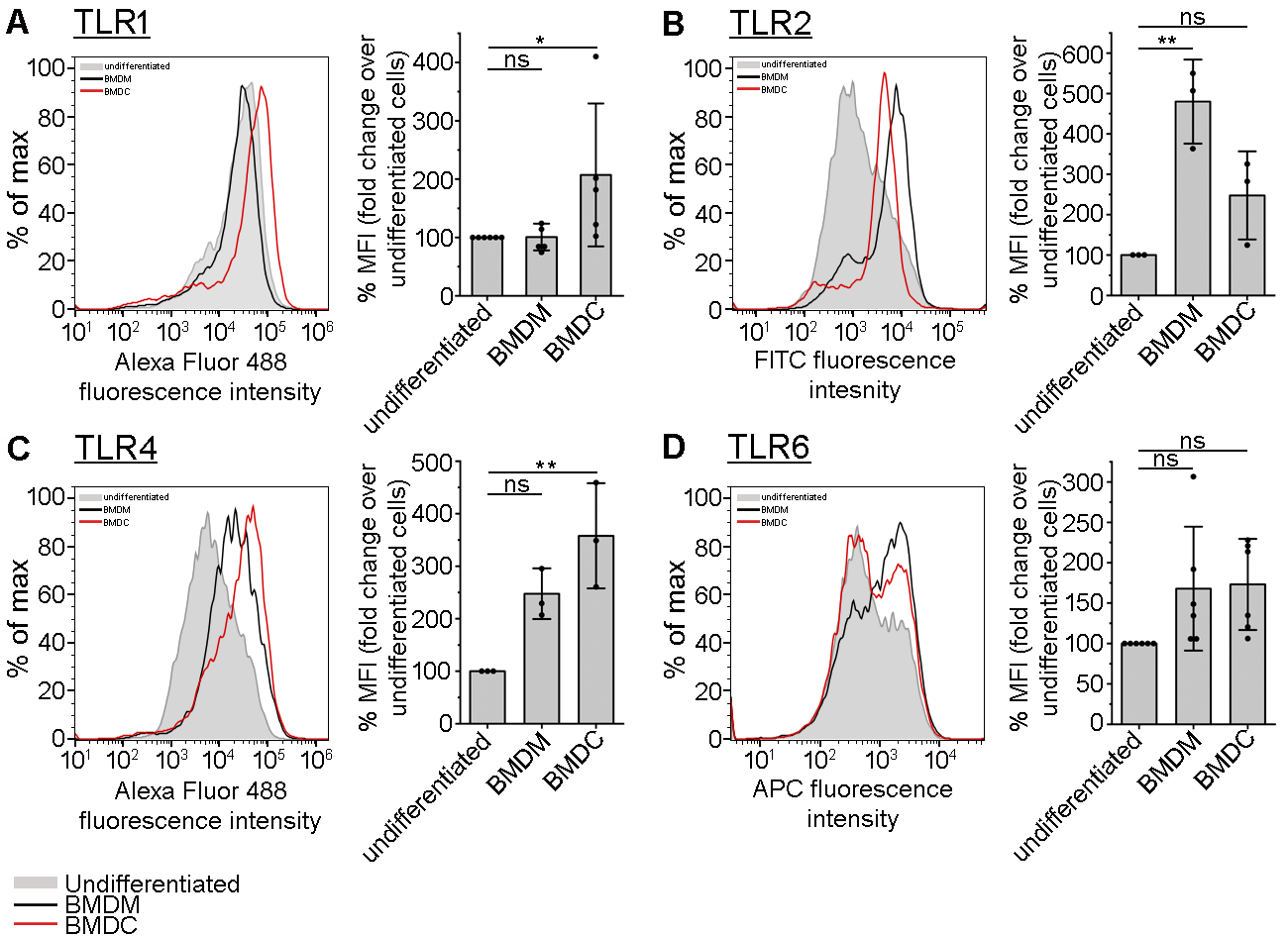

Supplement: S5 Fig — Differentiation of the bone marrow-derived cells into BMDMs led to a prominent increase in the surface expression of TLR2, TLR4, as well as TLR6, and no noticeable increase in the TLR1 surface expression. In contrast, differentiation into the BMDCs induced a significant increase in the surface expression of TLR1 and TLR4, and some moderate increase in the TLR2 and TLR6. Flow cytometry-based assay was employed to probe the surface expressions of different TLRs, and the representative histogram plots are shown (A-D). Extent of surface expression was quantitated by calculating the %Median Fluorescence Intensity (%MFI) with respect to that from the undifferentiated cells (corresponding to 100%), and the averages ± SDs (from three to six independent experiments) are shown in the form of bar graphs (A-D). ns, non-significant; *, p < 0.05; **, p < 0.01; one-way ANOVA with Dunnet’s multiple comparison test. (TIF) [file ppat.1013033.s005.tif]

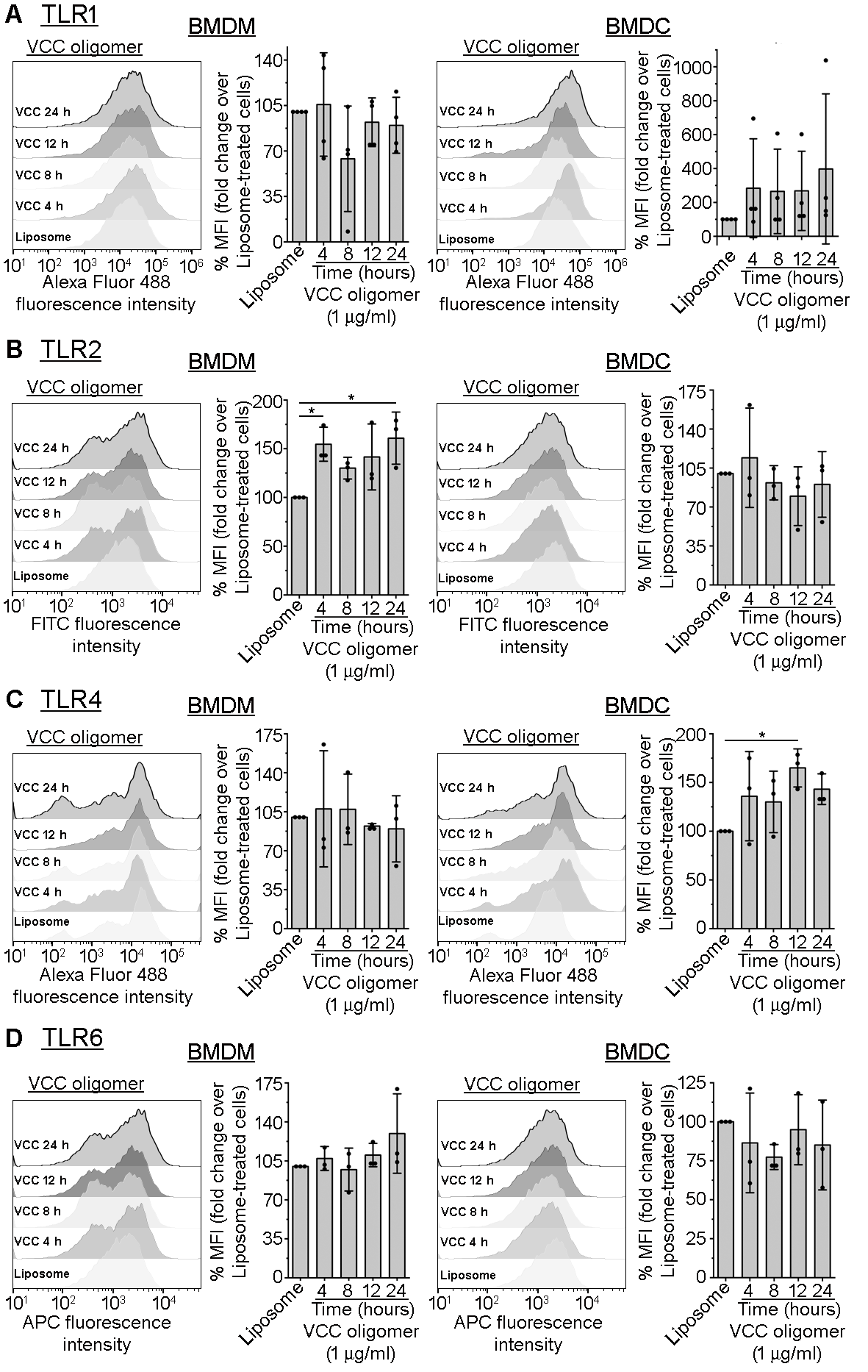

Supplement: S6 Fig — (A-D) Flow cytometry-based data showing changes, if any, in the surface expression of TLR1 (A), TLR2 (B), TLR4 (C), and TLR6 (D) upon treatment of BMDMs or BMDCs with VCC oligomer (1 μg/ml; for the specified time periods). Subsequently, TLR expressions were monitored by flow cytometry. Liposome-treated cells served as the negative controls. Offset histograms (shown in A-D) are the representatives of three to four independent experiments. Extent of TLR surface expressions was quantitated by calculating the %Median Fluorescence Intensity (%MFI) with respect to that from the liposome-treated cells (corresponding to 100%), and the averages ± SDs (from three to four independent experiments) are shown in the form of bar graphs (A-D). *, p < 0.05; one-way ANOVA with Dunnet’s multiple comparison test. (TIF) [file ppat.1013033.s006.tif]

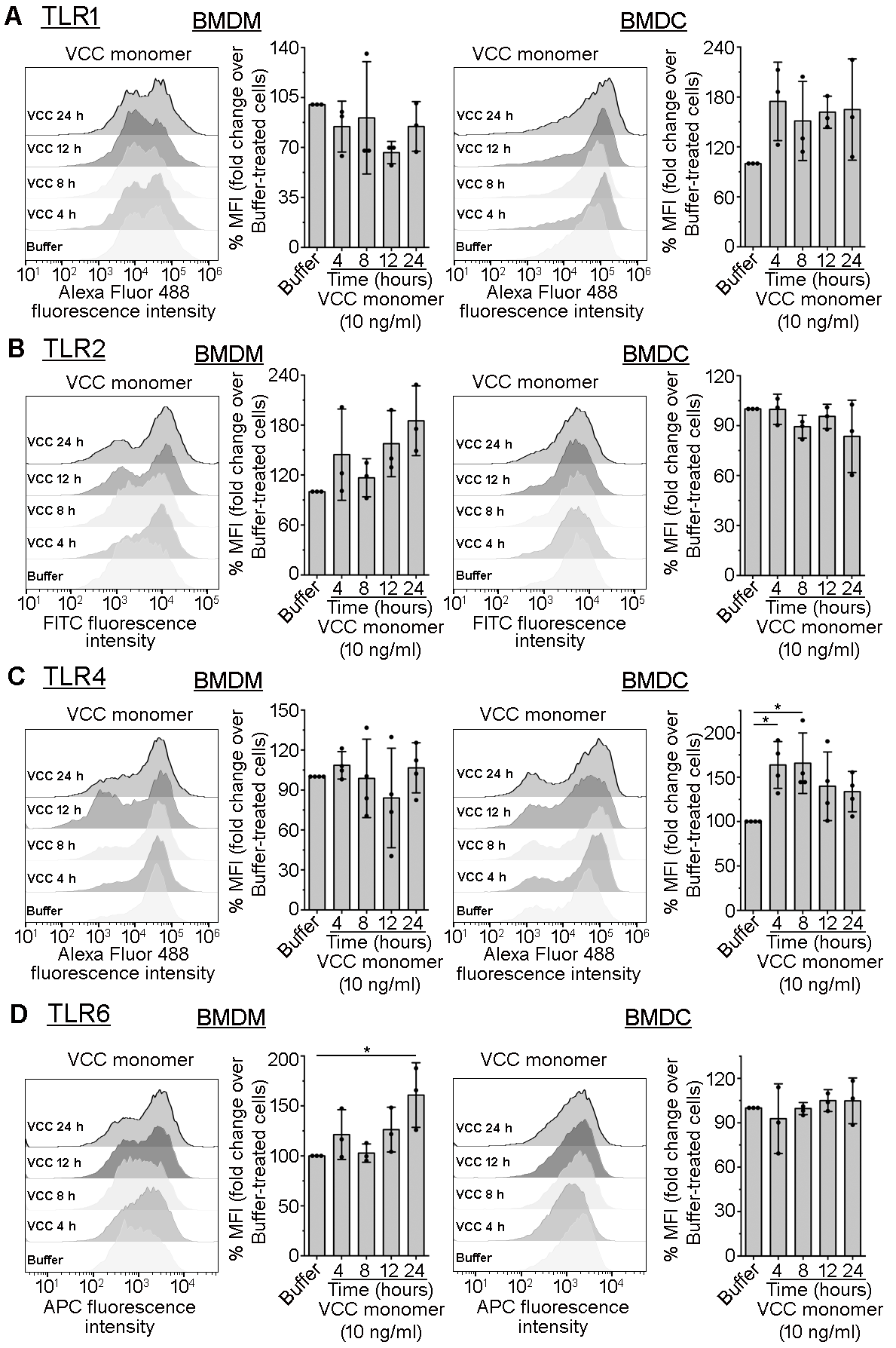

Supplement: S7 Fig — (A-D) Flow cytometry-based data showing changes, if any, in the surface expression of TLR1 (A), TLR2 (B), TLR4 (C), and TLR6 (D) upon treatment of BMDMs or BMDCs with VCC monomer (10 ng/ml; for the specified time periods). Subsequently, TLR expressions were monitored by flow cytometry. Buffer-treated cells served as the negative controls. Offset histograms (shown in A-D) are the representatives of three to four independent experiments. Extent of TLR surface expressions was quantitated by calculating the %Median Fluorescence Intensity (%MFI) with respect to that from the buffer-treated cells (corresponding to 100%), and the averages ± SDs (from three to four independent experiments) are shown in the form of bar graphs (A-D). *, p < 0.05; one-way ANOVA with Dunnet’s multiple comparison test. (TIF) [file ppat.1013033.s007.tif]

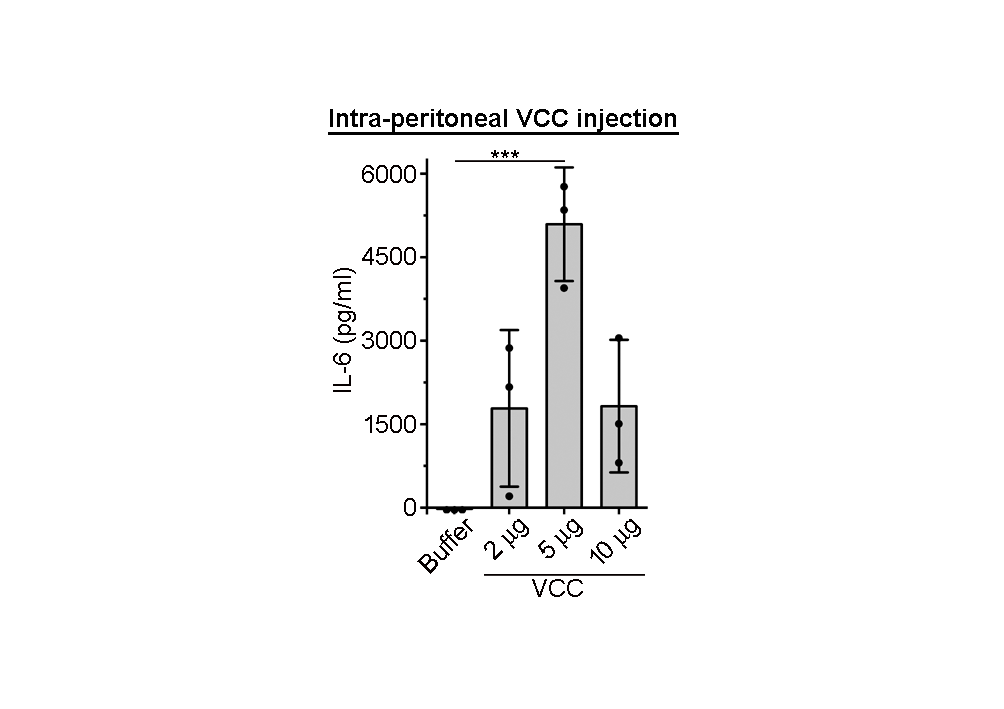

Supplement: S8 Fig — C57BL/6 mice were injected intra-peritoneally with 2, 5, and 10 μg VCC (in 100 μl PBS), and blood was collected after 4 hours. Serum was obtained, and IL-6 level in the serum was estimated by ELISA. Mice injected with equal volume of PBS served as the negative controls. Data shown here are the averages ± SDs from three independent experiments. ***, p < 0.001; one-way ANOVA with Dunnett’s multiple comparison test. (TIF) [file ppat.1013033.s008.tif]
